# Discovery of PRMT3 Degrader for the Treatment of Acute Leukemia

**DOI:** 10.1002/advs.202405963

**Published:** 2024-08-09

**Authors:** Wanyi Zou, Mengna Li, Shili Wan, Jingkun Ma, Linan Lian, Guanghao Luo, Yubo Zhou, Jia Li, Bing Zhou

**Affiliations:** ^1^ State Key Laboratory of Drug Research, Shanghai Institute of Materia Medica Chinese Academy of Sciences 555 Zu Chong Zhi Road Shanghai 201203 China; ^2^ University of Chinese Academy of Sciences 19 Yuquan Road Beijing 100049 China; ^3^ School of Chinese Materia Medica Nanjing University of Chinese Medicine Nanjing 210023 China; ^4^ Zhongshan Institute for Drug Discovery, Shanghai Institute of Materia Medica Chinese Academy of Sciences Guangdong 528400 China; ^5^ Shandong Laboratory of Yantai Drug Discovery Bohai Rim Advanced Research Institute for Drug Discovery Yantai Shandong 264117 China; ^6^ School of Pharmaceutical Science and Technology, Hangzhou Institute for Advanced Study University of Chinese Academy of Sciences Hangzhou 310024 China

**Keywords:** acute leukemia, MDM2, oxidative phosphorylation, PRMT3, PROTAC

## Abstract

Protein arginine methyltransferase 3 (PRMT3) plays an important role in gene regulation and a variety of cellular functions, thus, being a long sought‐after therapeutic target for human cancers. Although a few PRMT3 inhibitors are developed to prevent the catalytic activity of PRMT3, there is little success in removing the cellular levels of PRMT3‐deposited ω‐N^G^,N^G^‐asymmetric dimethylarginine (ADMA) with small molecules. Moreover, the non‐enzymatic functions of PRMT3 remain required to be clarified. Here, the development of a first‐in‐class MDM2‐based PRMT3‐targeted Proteolysis Targeting Chimeras (PROTACs) **11** that selectively reduced both PRMT3 protein and ADMA is reported. Importantly, **11** inhibited acute leukemia cell growth and is more effective than PRMT3 inhibitor **SGC707**. Mechanism study shows that **11** induced global gene expression changes, including the activation of intrinsic apoptosis and endoplasmic reticulum stress signaling pathways, and the downregulation of E2F, MYC, oxidative phosphorylation pathways. Significantly, the combination of **11** and glycolysis inhibitor **2‐DG** has a notable synergistic antiproliferative effect by further reducing ATP production and inducing intrinsic apoptosis, thus further highlighting the potential therapeutic value of targeted PRMT3 degradation. These data clearly demonstrated that degrader **11** is a powerful chemical tool for investigating PRMT3 protein functions.

## Graphical abstract

The discovery of a first‐in‐class PRMT3‐targeted PROTAC **11** that effectively and selectively induced PRMT3 degradation is described by recruiting MDM2 E3 ubiquitin ligase. Importantly, **11** effectively inhibited the acute leukemia cell growth and is more effective than PRMT3 inhibitor **SGC707**, highlighting the advantages of PRMT3 degradation over inhibition.

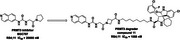

## 1 Introduction

Arginine methylation was first discovered and defined by Paik and Kim >50 years ago.^[^ ^1^ ^]^ The process of arginine methylation is regulated by arginine methyltransferases (PRMTs), which perform their functions by transferring the methyl group from the S‐5′‐adenosyl‐L‐methionine (SAM) to the arginine residues of protein arginine tails.^[^ ^2^ ^]^ By now, nine different isoforms of PRMTs have been identified and can be classified into three categories according to their methylation functions, which are monomethylation, asymmetric dimethylation, and symmetric dimethylation. PRMT3 is one of the type I PRMTs that catalyzes mono‐ and asymmetric arginine dimethylation.^[^ ^3^ ^]^ By now, numerous evidences have demonstrated that PRMT3 is able to regulate the methylation of glyceraldehyde‐3‐phosphate dehydrogenase (GAPDH), lactate dehydrogenase A (LDHA), and MYC to participate in the metabolic reprogramming of tumorigenesis.^[^ ^4^ ^]^ Numerous studies also revealed the key role of PRMT3 in multiple kinds of pathological processes, such as hematological malignancies,^[^ ^5^ ^]^ gemcitabine (GEM)‐resistant pancreatic cancer,^[^ ^6^ ^]^ hepatocellular carcinoma,^[^ ^4b^ ^]^ colorectal cancer,^[^ ^7^ ^]^ and breast cancer.^[^ ^8^ ^]^ Therefore, PRMT3 is potentially an attractive therapeutic target for human cancers.

Although a few PRMT3 inhibitors have been developed to prevent the catalytic activity of PRMT3,^[^ ^9^ ^]^ the inhibitors were not effective in removing the PRMT3‐deposited ADMA. Furthermore, the biological functions of PRMT3, especially non‐enzymatic functions in cancer cell lines remains required to be clarified. We speculated that only inhibition of the enzymatic function of PRMT3 might be insufficient to result in a total loss‐of‐function. Therefore, there is an unmet need to develop alternative strategies to target PRMT3.

In recent years, Proteolysis Targeting Chimeras (PROTACs) strategy has emerged as a novel and attractive therapeutic approach that degrade rather than inhibit target proteins.^[^ ^10^ ^]^ Notably, unlike classic inhibitors, PROTAC has the advantage of simultaneously regulating the enzymatic and non‐enzymatic protein functions,^[^ ^11^ ^]^ thus providing a potential strategy to compensate for the shortcoming of inhibitors. To the best of our knowledge, no PRMT3 PROTAC degraders have been reported to date.

In this context, we described the discovery of a first‐in‐class PRMT3 selective degrader **11** by recruiting MDM2 E3 ubiquitin ligase that induced PRMT3 degradation in acute leukemia (AL) cells with a DC_50_ value of 2.5 µM. Importantly, degrader **11** exhibited more effective cell growth inhibition and especially much more significant decrease of ADMA than PRMT3 inhibitor **SGC707**. More significantly, degrader **11** in combination with glycolysis inhibitor 2‐Deoxy‐D‐glucose (**2‐DG**) displayed notable synergistic anti‐proliferative activities, whereas no synergistic effect was observed in the **2‐DG** and inhibitor **SGC707** combination group, further highlighting the advantages of PRMT3 degradation over inhibition.

## 2 Results and Discussion

**Figure 1:**
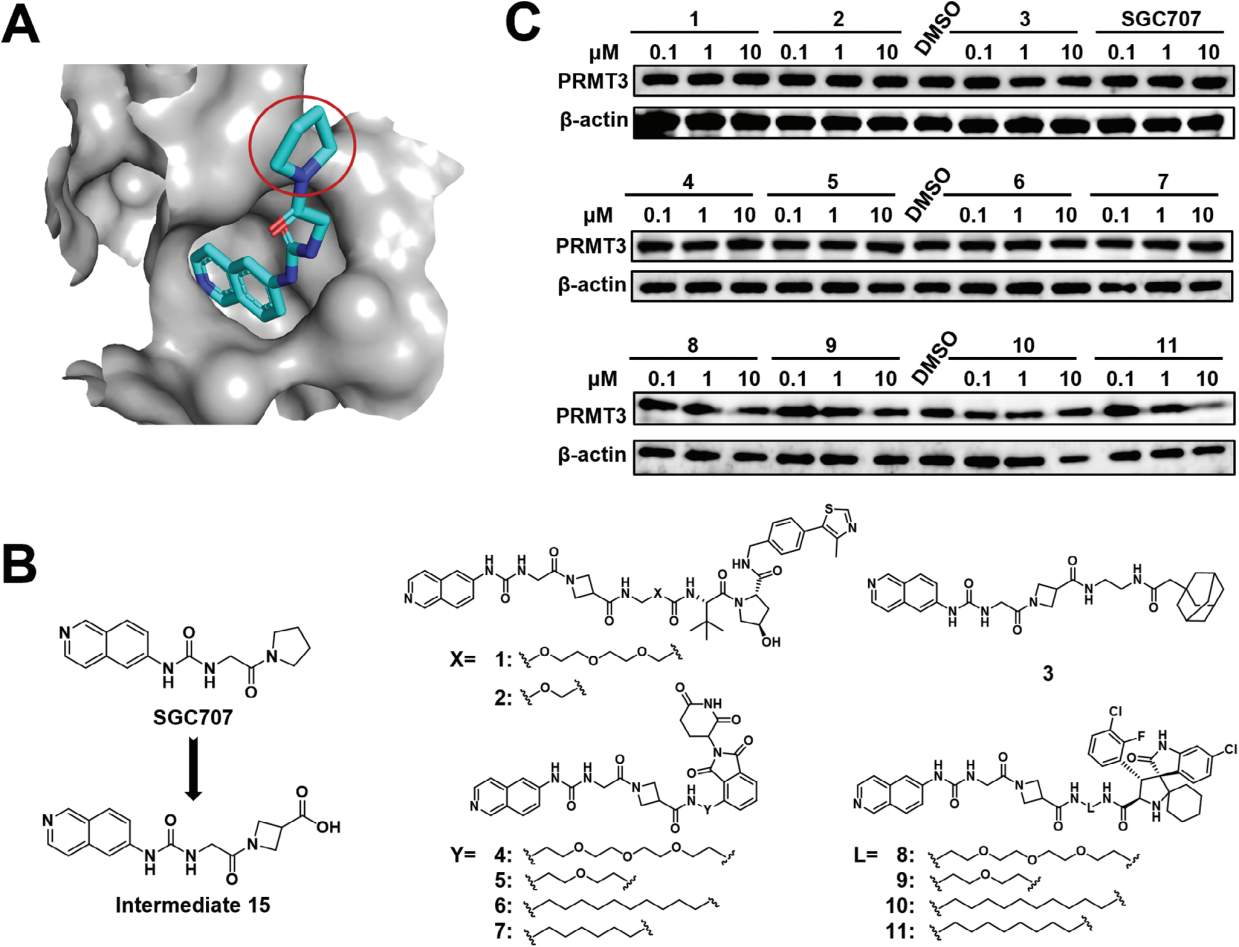
Design of PRMT3 PROTACs. A) The co‐crystal structure of PRMT3 (in gray) in complex with **SGC707** (PDB: 4RYL). B) Design of PRMT3 degraders based on the PRMT3 inhibitor **SGC707**. C) Immunoblot analysis for PRMT3 in RS4;11 cells treated with the indicated compounds for 24 h.

To develop PRMT3 PROTACs, PRMT3 inhibitor **SGC707** was chosen as a PRMT3‐targeting ligand.^[^ ^9b^ ^]^ The co‐crystal structure of **SGC707** with PRMT3 reveals that the pyrrolidinyl group is oriented toward the solvent and is a suitable tethering site to design PROTACs (**Figure** **1**A). We first converted the pyrrolidinyl group to a 3‐azetidinecarboxylic acid to give the intermediate **15** for facilitating linker installation (Figure 1B). Subsequently, PROTACs with different E3 ligase ligands such as Von Hippel‐Lindau (VHL) ligand (**1** and **2**), cereblon (CRBN) ligand (**4‐7**) and MDM2 ligand (**8‐11**), as well as hydrophobic tagging (**3**) were synthesized and tested (Figure 1C). Surprisingly, VHL (**1** and **2**) and CRBN ligands (**4**‐**7**) that have been employed in the majority of reported PROTAC molecules and were prove to be two of the most effective PROTAC E3 ligase ligands, failed to induce PRMT3 degradation in RS4;11 cells (Figure 1C). Compound **3** with hydrophobic tagging was also ineffective (Figure 1C). Interestingly, PROTACs **8**–**11** bearing a MDM2 ligand with weak binding affinity to MDM2.^[^ ^12^ ^]^ induced effective degradations of PRMT3 at 10 µm in RS4;11 cells. The potency of the prepared molecules for PRMT3 degradation in MV‐4‐11 cell lines was also evaluated (Figure S1B, Supporting Information). Similar results were observed and only MDM2‐recruiting PROTACs **8**–**11** were capable of inducing the PRMT3 degradation. On the basis of the analysis of the western blots in RS4;11 and MV‐4‐11 cells, compound **11** stood out as the most potent degrader of PRMT3 and was selected for further evaluation.

**Figure 2:**
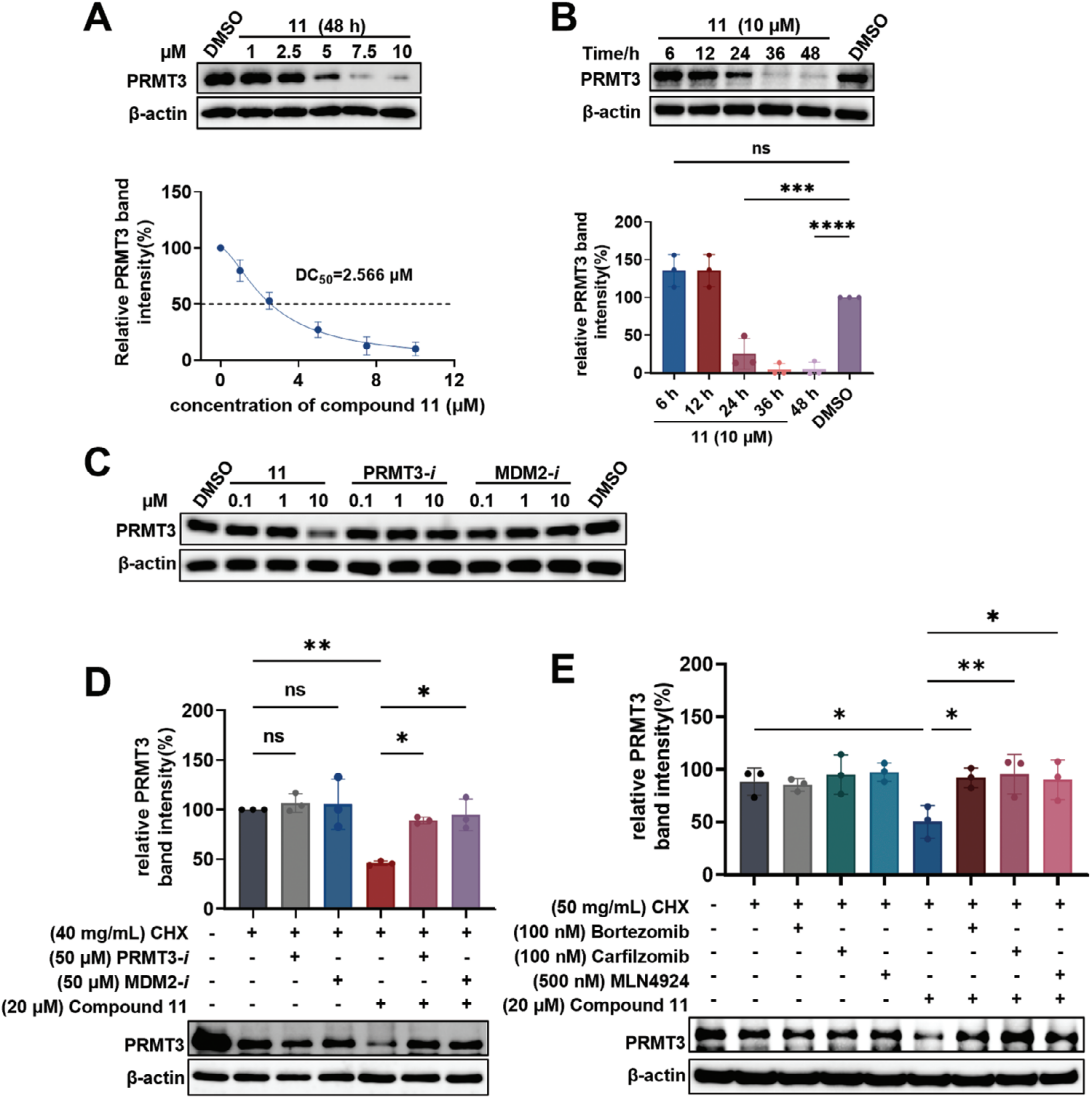
Degradation profiling of **11**. A) Immunoblots for PRMT3 in RS4;11 cells after treatment with the indicated concentrations of **11** for 48 h and determination of DC_50_ and D_max_ value of **11**. The gray values of PRMT3 protein bands were calculated with Image Lab 6.0. Error bars represent mean ± SD from n = 3 independent replicates. B) Immunoblots for PRMT3 in RS4;11 cells treated with 10 µm of **11** at indicated time points. The gray values of PRMT3 protein bands were calculated with Image Lab 6.0. Error bars represent mean ± SD from n = 3 independent replicates. C) Immunoblots PRMT3 in RS4;11 cells treated with indicated concentrations of **11**, **PRMT3‐*i*** and **MDM2‐*i*** for 24 h. D) Immunoblots for PRMT3 in RS4;11 cells pre‐treated with DMSO, 50 µm of **PRMT3‐*i***, or 50 µm of **MDM2‐*i*** for 2 h, and co‐treated with 20 µm of **11** for 12 h in the presence of 40 mg mL^−1^ of CHX. The gray values of PRMT3 protein bands were calculated with Image Lab 6.0. Error bars represent mean ± SD from n = 3 independent replicates. E) Immunoblots for PRMT3 in RS4;11 cells pre‐treated with DMSO, 100 nm of Bortezomib, 100 nm of Carfilzomib or 500 nm of MLN4924 for 2 h, and then co‐treated with 20 µm of **11** for 9 h in the presence of 50 mg mL^−1^ CHX in RS4;11 cells. The gray values of PRMT3 protein bands were calculated with Image Lab 6.0. Error bars represent mean ± SD from n = 3 independent replicates. *P* values (B, D, E) were calculated by one‐way analysis of variance (ANOVA). Significance levels are as follows: ^*^ *p* < 0.05, ^**^ *p* < 0.01, ^***^ *p* < 0.001, ^****^ *p* < 0.0001; ns indicates non‐significant.

The degradation efficacy of **11** on PRMT3 was next evaluated in RS4;11 cell lines. As shown in **Figure** **2**A, **11** effectively induced PRMT3 degradation in RS4;11 cell lines in a dose‐dependent manner with a DC_50_ (half‐maximal degradation) value of 2.5 µm and a D_max_ of 90%, while the levels of other isoforms PRMT5 and PRMT6 remained unaffected (Figure S1C, Supporting Information). Similar dose‐dependent PRMT3 degradation in MOLM13 and MOLT4 cell lines was also observed (Figure S1D, Supporting Information). A time course experiment revealed that **11** effectively degraded PRMT3 in RS4;11 cells after 24 h of exposure (Figure 2B).

To explore the mechanism of PRMT3 degradation, several experiments were performed. Neither the PRMT3 ligand **PRMT3‐*i*** nor the MDM2 ligand **MDM2‐*i*** was able to induce PRMT3 degradation (Figure 2C; Figure S2A, Supporting Information, for structures). **11** had no influence on the mRNA levels of PRMT3 in RS4;11 cells (Figure S2B, Supporting Information) and the efficacy of PRMT3 degradation induced by **11** was increased by using cycloheximide (CHX) chase assay (Figure S2C, Supporting Information). Furthermore, the addition of PRMT3 inhibitor **PRMT3‐*i*** or MDM2 binder **MDM2‐*i*** could rescue PRMT3 degradation (Figure 2D). Taken together, these results demonstrated that the PRMT3‐11‐MDM2 ternary complex formation is necessary for PRMT3 degradation. Additionally, pre‐treatment of NEDD8‐activating enzyme inhibitor MLN4924 or proteasome inhibitor Bortezomib and Carfilzomib effectively blocked the degradation of PRMT3 by **11**, suggesting a proteasomal and neddylation dependent substrate degradation (Figure 2E).

**Figure 3:**
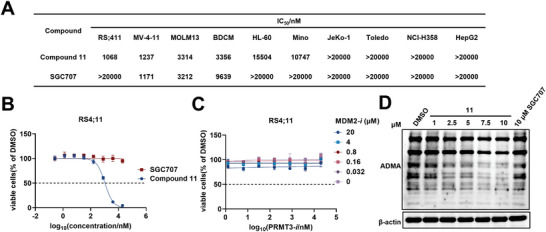
Compound **11** showed cell growth inhibitory activities. A) Cell growth inhibitory activities of **SGC707** and **11** in cancer cells, among which RS4;11, MV‐4‐11, MOLM13, BDCM, and HL‐60 are AL cell lines. B) Cell growth inhibitory activities of **11** and **SGC707** in RS4;11 cells for 3 days using cell counting kit‐8 (CCK8). Plotted as mean ± SD for n = 3 replicates. C) Cell growth inhibitory activities of the combination of **MDM2‐*i*** and **PRMT3‐*i*** in RS4;11 cells for 3 days. Plotted as mean ± SD for n = 3 replicates. D) Analysis of ADMA in RS4;11 cells treated with indicated concentrations of **11** and **SGC707**.

To probe the phenotypic effects of PRMT3 degradation versus PRMT3 inhibition, we first evaluated the anti‐proliferative effect of **11** and inhibitor **SGC707** in a panel of cancer cells including AL, lymphoma, and solid tumor cell lines. Overall, **11** showed more potent anti‐cancer effect than **SGC707**. Among the cell lines we tested, the AL cells were more sensitive than other cancer cells to **11** (**Figure** **3**A). Significantly, **11** could effectively inhibit the cell growth in a dose‐dependent manner in RS4;11 cells, whereas the inhibitor **SGC707** had no effect (Figure 3B). The combination of PRMT3 inhibitor **PRMT3‐*i*** with MDM2 ligand **MDM2‐*i*** did not exhibit cell growth inhibitory activity in RS4;11 cells (Figure 3C), suggesting that the cell growth inhibition induced by **11** was caused by PRMT3 degradation, not by the addition or synergistic effects of PRMT3 inhibition and MDM2 inhibition. To further verify that the cell growth inhibitory activity of **11** is PRMT3 degradation dependent, we obtained RS4;11 PRMT3 knockdown cells (Figure S3A, Supporting Information) and found that **11** had significantly lost its cell growth inhibitory activity in RS4;11 PRMT3 knockdown cells (Figure S3B, Supporting Information). The primary function of PRMT3 is to transfer methyl group from the methyl donor S‐adenosylmethionine (SAM) to the terminal nitrogen of arginine residue to form ω‐N^G^,N^G^‐asymmetric dimethylarginine (ADMA). Therefore, global cellular levels of ADMA were subsequently assessed. As shown in Figure 3D, **11** resulted in a significant decrease of ADMA in a dose‐dependent manner, while the PRMT3 inhibitor **SGC707** showed a minimal down‐regulation of ADMA even at a concentration of 10 µm. Taken together, these results revealed that degrader **11** showed much more potent anti‐cancer effect and more significant ADMA decrease than PRMT3 inhibitor.

**Figure 4:**
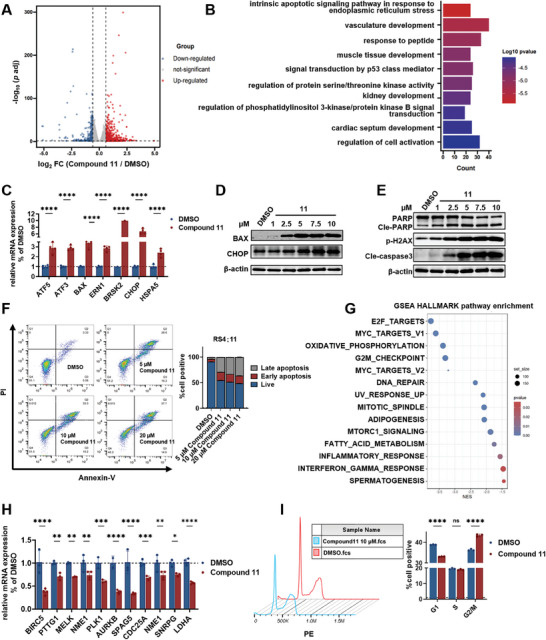
Mechanism study of degrader **11**. A) Volcano plots of differentially expressed genes (DEGs) in RS4;11 cells after treatment with 10 µm of **11** for 24 h, relative to DMSO (n = 3). Genes with log2‐fold change between **11** and DMSO treatment samples >0.585 or ←0.585 were classified as DEGs and the adjusted P‐value (adj P‐value) <0.05 was considered as statistical significance. B) GO analysis of up‐regulated genes in RS4;11 cells treated with **11** compared to DMSO. C) Real‐time qPCR analysis of intrinsic apoptosis signaling pathway in response to endoplasmic reticulum stress‐related genes normalized to the expression of VCL in RS4;11 cells. Error bars represent mean ± SD from n = 3 replicates. D) Immunoblot for BAX and CHOP in RS4;11 cells treated with **11** at the indicated concentration for 48 h. E) Immunoblot for cleaved PARP, caspase 3, and p‐H2AX in RS4;11 cells treated with **11** at indicated concentration for 48 h. F) Flow cytometry analysis of cell apoptosis in RS4;11 cells treated with **11** at indicated concentration or with DMSO for 24 h. Error bars represent mean ± SD from n = 3 replicates. G) HALLMARK GSEA analysis of the down‐regulated genes in RS4;11 cells treated with **11**. H) Real‐time qPCR analysis. Error bars represent mean ± SD from n = 3 replicates. I) The effects of **11** on cell circle was analyzed at 24 h after treatment with 10 µm of **11** and DMSO in PI‐stained nuclei using FlowJo software. Error bars represent mean ± SD from n = 3 replicates. *P* values (C, H, I) were calculated by two‐way analysis of variance (ANOVA). Significance levels are as follows: ^*^ *p* < 0.05, ^**^ *p* < 0.01, ^***^ *p* < 0.001, ^****^ *p* < 0.0001; ns indicates non‐significant.

To gain further insights into understanding how PRMT3 degradation result in antiproliferative activities, transcriptome interrogation was performed. As shown in **Figure** **4**A, compound **11** caused 687 genes to be up‐regulated and 290 genes to be down‐regulated compared with the control. Further Gene set enrichment analysis (GSEA) revealed that **11** led to significant changes in several important cancer cell survival pathways. As shown in Figure 4B, the upregulated genes were mainly enriched in intrinsic apoptosis signaling pathways, endoplasmic reticulum stress, vasculature development, and regulation of protein serine/threonine kinase activity. Subsequent reverse transcription followed by quantitative polymerase chain reaction (RT‐qPCR) experiment verified that **11** significantly up‐regulated the expression of intrinsic apoptotic signaling pathway and endoplasmic reticulum stress‐related genes (Figure 4C). Immunoblotting further confirmed that **11** upregulated BAX and CHOP in a dose‐dependent manner (Figure 4D). Additionally, treatment with **11** also led to the upregulation of cleaved caspase‐3 and cleaved PARP as well as γH2AX, a sensitive DNA damage maker in a dose‐dependent manner (Figure 4E). Consistently, **11** caused significant induction of apoptosis in RS4;11 cells (Figure 4F). GSEA also revealed that several important cancer cell survival pathways, such as the E2F, MYC, oxidative phosphorylation, and G2M checkpoint pathways were significantly down‐regulated by **11** (Figure 4G). RT‐qPCR experiment further confirmed that the mRNA expression of E2F, MYC, and cell cycle related genes was down‐regulated (Figure 4H). FACS assay showed that **11** obviously induced cell cycle G2/M arrest (Figure 4I). Taken together, these results clearly showed that the **11** treatment could induce cell apoptosis and cell cycle arrest that might be related to the changes of endoplasmic reticulum stress as well as E2F and MYC pathways.

**Figure 5:**
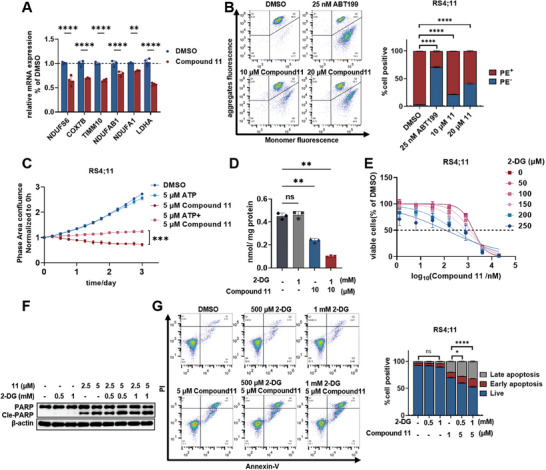
Synergistic effect study of **11** and **2‐DG**. A) Real‐time qPCR analysis of the oxidative phosphorylation‐related genes normalized to the expression of VCL in RS4;11 cells. Error bars represent mean ± SD from n = 3 independent replicates. B) Flow cytometry analysis of mitochondrial membrane potential of RS4;11 cells treated with **11** and ABT‐199 at indicated concentration for 24 h. Error bars represent mean ± SD from n = 2 independent replicates. C) Cell growth inhibitory activities of 5 µm of **11** alone, 5 µm of ATP alone, and combination treatment monitored by IncuCyte. Mean ± SD is shown (n = 3). D) Intracellular ATP in RS4;11 treated with **2‐DG**, **11** alone or combination at indicated concentration for 24 h. Error bars represent mean ± SD from n = 3 technical replicates. E) Cell growth inhibitory activities of the combination of **11** and **2‐DG** in RS4;11 cells. F) Immunoblot for cleaved PARP, and Cle‐caspase 3 in RS4;11 cells treated with **11**, **2‐DG**, or combination. G) Flow cytometry analysis of cell apoptosis (left) and quantification (right) in RS4;11 cells treated with **11**, **2‐DG**, or combination for 24 h. Error bars represent mean ± SD from n = 3 replicates. *P* values were calculated by two‐way analysis of variance (A, B, G), one‐way analysis of variance (D) and two‐tail Student's T‐tests (C). Significance levels are as follows: ^*^ *p* < 0.05, ^**^ *p* < 0.01, ^***^ *p* < 0.001, ^****^ *p* < 0.0001; ns indicates non‐significant.

To further verify if the oxidative phosphorylation pathway was down‐regulated by **11**, real‐time qPCR experiment was performed, showing that **11** resulted in the down‐regulation of a panel of genes related with oxidative phosphorylation in RS4;11 cells (**Figure** **5**A). Mitochondria is where oxidative phosphorylation takes place and encodes a variety of subunit proteins that are critical to the function of oxidative phosphorylation.^[^ ^13^ ^]^ Mitochondrial membrane potential (MtMP) is a key indicator of mitochondrial activity and it reflects the process of electron transport and oxidative phosphorylation, the driving force behind ATP production.^[^ ^14^ ^]^ Therefore, the mitochondrial membrane potential was subsequently examined and BCL‐2 inhibitor ABT‐199 was used as a positive control. As expected, treatment of **11** led to the loss of mitochondrial membrane potential and mitochondrial impairment (Figure 5B). To establish convincingly that the antiproliferative effect of **11** was caused by a decrease of oxidative phosphorylation, we examined the antiproliferative activities of **11** in the presence of exogenous ATP in the IncuCyte S5 platform. The addition of exogenous ATP could significantly reverse the cell growth inhibitory activity of **11** (Figure 5C). This reversal effect was also observed in apoptosis as early as 24 h when exogenous ATP was added (Figure S4A,B, Supporting Information). These results revealed that the treatment of **11** resulted in a decrease of oxidative phosphorylation, which participated in its antiproliferative effect, at least partly.

Inhibition both of glycolysis and oxidative phosphorylation could lead to a significant synergetic effect in cancer treatments.^[^ ^15^ ^]^ Therefore, we subsequently performed a drug combination experiment to determine the potential synergistic effect of degrader **11** and glycolysis inhibitor **2‐DG** in RS4;11 cells. Excitingly, the combination of **11** and **2‐DG** markedly inhibited ATP production to lower levels than that seen with either single‐agent treatment, although **2‐DG** had no influence on ATP decrease (Figure 5D). Moreover, the addition of **2‐DG** effectively decreased the ATP production in PRMT3 knockdown cells (Figure S4C, Supporting Information). Consistent with ATP decrease, a strong synergistic cell growth inhibition was observed in the **2‐DG** and **11** combination group (Figure 5E). In keeping with these results, the cleaved PARP and the ratio of cell apoptosis were further prominently increased in the **11** and **2‐DG** combination group (Figure 5F,G). In addition, similar synergistic results were obtained for MV‐4‐11 and MOLM13 cells (Figure S4D, Supporting Information). Notably, no synergistic effect on ATP production and cell growth inhibition were observed in the **2‐DG** and inhibitor **SGC707** combination group (Figure S4E,F, Supporting Information), further highlighting the advantages of PRMT3 degradation over inhibition. Overall, these results revealed that glycolysis inhibitor **2‐DG** was capable of sensitizing AL cells to PRMT3 degrader **11** by further reducing ATP production and inducing intrinsic apoptosis.

Finally, a preliminary pharmacokinetics (PK) and pharmacodynamics (PD) studies were performed using mice models. Administration of degrader **11** via both intraperitoneal and intravenous injection exhibited substantial plasma drug exposure and good bioavailability (Table S12, Supporting Information). Next, PD studies were carried out and degrader **11** was administered to mice via intraperitoneal injection. As shown in Figure S5 (Supporting Information), the results showed that intraperitoneal injection of degrader **11** resulted in significant PRMT3 degradation observed 32 h after treatment in mouse models.

## 3 Conclusion

PRMT3 has been a long sought‐after therapeutic target for human cancers, however, efforts to manipulate both PRMT3 activity and ADMA levels via chemical approaches have been largely unsuccessful. Herein, we developed a first‐in‐class PRMT3 degrader **11** by recruiting MDM2 E3 ubiquitin ligase that induced PRMT3 degradation with a DC_50_ value of 2.5 µm and a D_max_ of 90%. Notably, degrader **11** exhibited more effective cell growth inhibition and more significant ADMA decrease than inhibitor **SGC707** in several AL cell lines. Further mechanism study revealed that the PRMT3 degradation induced by **11** was involved in several important cancer cell survival pathways, including the activation of intrinsic apoptosis and endoplasmic reticulum stress signaling pathways, and the downregulation of E2F, MYC, oxidative phosphorylation, G2M checkpoint pathways.

Additionally, the down‐regulation of oxidative phosphorylation after **11** treatment was further confirmed, which promoted us to further investigate the potential synergistic effect of **11** and glycolysis inhibitor **2‐DG**. Significantly, the combination of **11** and **2‐DG** led to a notably enhanced antiproliferative effect in AL cells with further reduced ATP and increased intrinsic apoptosis, while the combination of **2‐DG** and inhibitor **SGC707** had no synergistic effect, thus further expanding the applicability of **11** and also highlighting the advantages of PRMT3 degradation over inhibition. As PRMT3 degraders may have unparalleled benefits over conventional PRMT3 inhibitors in therapeutic treatment, our data suggest that **11** can be a promising chemical probe for PRMT3‐related disease research, in addition to its potential use for the treatment of AL.

## 4 Experimental Section

### Animal Ethics Statement

All animal experiments conducted in the manuscript were approved by the Institutional Animal Care and Use Committee (IACUC) at Shanghai Institute of Materia Medica with approval number (IACUC: 2024‐01‐lj‐01).

### Statistics Analysis

The statistical analysis was performed using GraphPad Prism software (V9.5.1). For all experiments, the number of replications and the transformation or normalisation of the data are described in the corresponding legends and methods. Unless otherwise stated, results are presented as mean value ± SD. IC_50_ and DC_50_ values were obtained by performing non‐linear regression utilizing a variable slope (normalized response) in Graph Pad Prism (V9.5.1). Western blot band density was calculated by utilizing the Image Lab 6.0 software. Comparisons between two groups were conducted by Student's T‐tests and multiple comparisons were performed by one‐way or two‐way analysis of variance (ANOVA). Differences were considered statistically significant at a p value < 0.05. Significance levels are as follows: ^*^ *p* < 0.05, ^**^ *p* < 0.01, ^***^ *p* < 0.001, ^****^ *p* < 0.0001; ns indicates non‐significant. Additional details of the materials and methods can be found in the Supporting Information.

## Conflict of Interest

The authors declare no conflict of interest.

## Supporting information

Supporting Information

## Data Availability

The data that support the findings of this study are available on request from the corresponding author. The data are not publicly available due to privacy or ethical restrictions.
