# Supplementary material for: Discovery of PRMT3 Degrader for the Treatment of Acute Leukemia
Source: Adv Sci (Weinh). 2024 Aug 9;11(38):2405963. doi: 10.1002/advs.202405963 (PMC11481256; doi:10.1002/advs.202405963)
Supplement: Supplementary file 1 — Supporting Information [file ADVS-11-2405963-s001.pdf]

ADVANCED SCIENCE
Open Access

Supporting Information

for *Adv. Sci.*, DOI 10.1002/advs.202405963

Discovery of PRMT3 Degrader for the Treatment of Acute Leukemia

Wanyi Zou, Mengna Li, Shili Wan, Jingkun Ma, Linan Lian, Guanghao Luo, Yubo Zhou, Jia Li* and Bing Zhou**

Supporting Information

Discovery of PRMT3 Degrader for the Treatment of Acute Leukemia

Wanyi Zou[a,b,§], Mengna Li[c,a,§], Shili Wan[a], Jingkun Ma[a,b], Linan Lian [c,a], Guanghao Luo[a,b], Yubo Zhou[a,b,c, f*], Jia Li[a,b,c, d, e, f*], Bing Zhou[a,b,c,d,e,*]

[a] State Key Laboratory of Drug Research, Shanghai Institute of Materia Medica, Chinese Academy of Sciences, 555 Zu Chong Zhi Road, Shanghai, 201203, China.

[b] University of Chinese Academy of Sciences, 19 Yuquan Road, Beijing, 100049, China.

[c] School of Chinese Materia Medica, Nanjing University of Chinese Medicine, Nanjing 210023, China.

[d] Shandong Laboratory of Yantai Drug Discovery, Bohai Rim Advanced Research Institute for Drug Discovery, Yantai, Shandong, 264117, China.

[e] School of Pharmaceutical Science and Technology, Hangzhou Institute for Advanced Study, University of Chinese Academy of Sciences, Hangzhou 310024, China.

[f] Zhongshan Institute for Drug Discovery, Shanghai Institute of Materia Medica, Chinese Academy of Sciences, Guangdong, 528400, China.

[§] These authors contributed equally to this work.

Table of Contents

Supplementary Figures 3

Supplementary Tables 6

Supplementary Methods 10

Chemical Synthesis 17

NMR spectra of synthesized compounds 33

Reference 46

Supplementary Figures

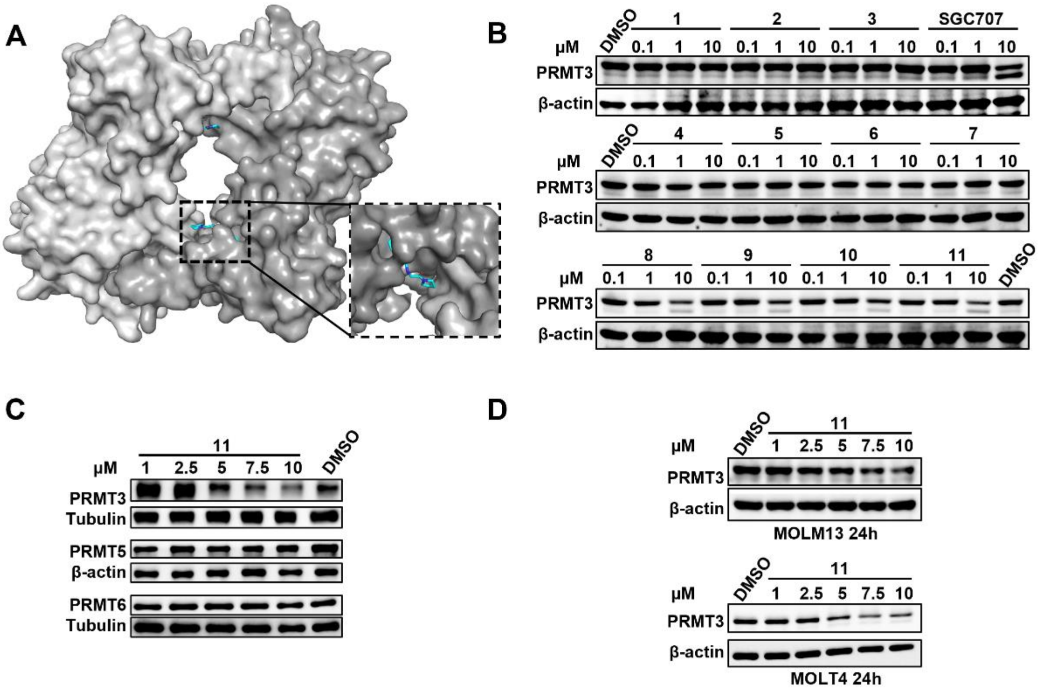

Figure S1. (A) The co-crystal structure of PRMT3 (in gray) dimer in complex with **SGC707** (in cyan). The binding site is emphasized. (B) Representative immunoblots evaluating total PRMT3 levels in MV-4-11 cells after treatment with the indicated compounds for 24 h. (C) Representative PRMT3, PRMT5, and PRMT6 immunoblot of RS4;11 cells treated with compound **11** at the indicated concentration for 24 h. (D) Representative immunoblots evaluating total PRMT3 levels in MOLM13, and MOLT4 cells after treatment with the indicated compounds for 24 h.

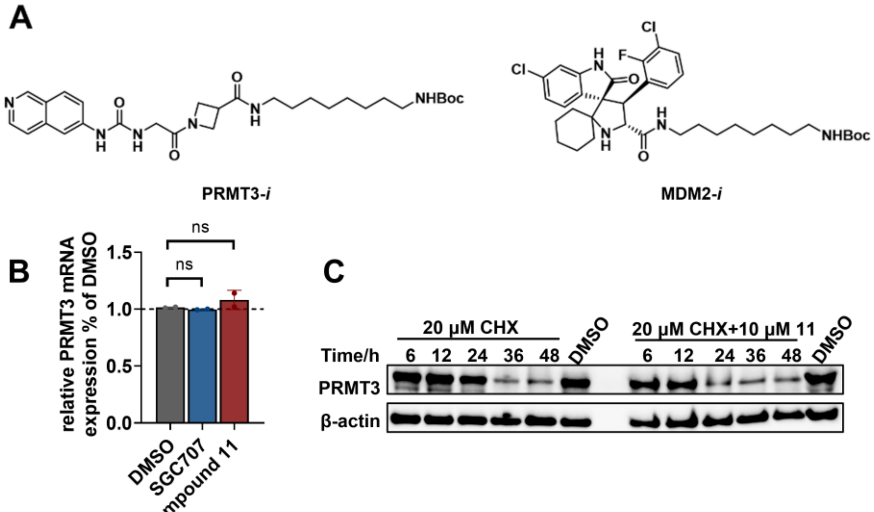

Figure S2. (A) Chemical structures of ***PRMT3-i*** and ***MDM2-i***. (B) Real-time qPCR analysis of mRNA levels of PRMT3 normalized to the expression of VCL in RS4;11 cells. Error bars represent

mean ± SD from n = 2 independent replicates. (C) Immunoblots of PRMT3 in RS4;11 cells treated with or without **11** in the presence of 20 μM of **cycloheximide** (CHX) for the indicated time. *P* values (B) were calculated by two-way analysis of variance. Significance levels are as follows: * p < 0.05, ** p < 0.01, *** p < 0.001, **** p < 0.0001; ns indicates non-significant.

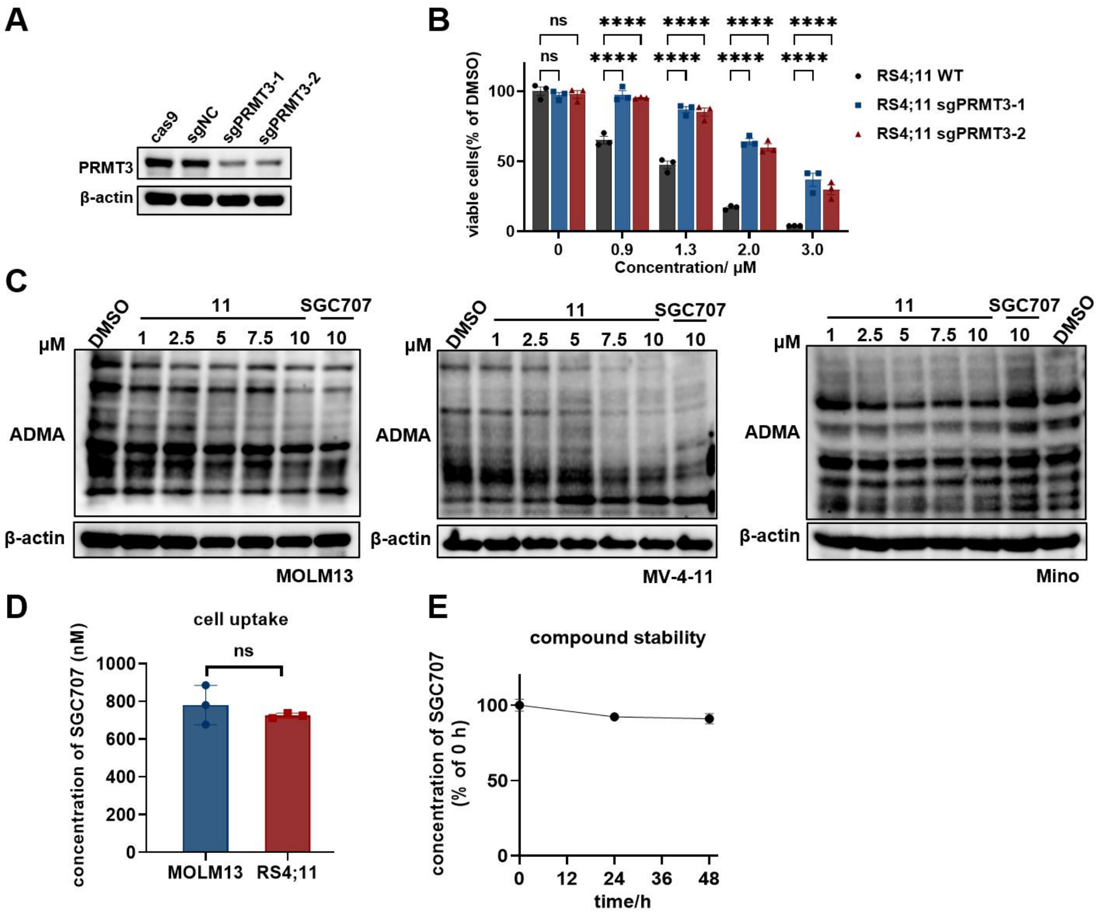

Figure S3. (A) Immunoblot validation of PRMT3 knockdown. (B) Growth curves of RS4;11 WT, sgPRMT3-1 and sgPRMT3-2 cells treated with the indicated concentrations of **11** for 72 h. Mean ± SD is shown (n = 3). (C) Analysis of ADMA in MOLM13, MV-4-11 and Mino cells treated with indicated concentrations of **11** and **SGC707**. (D) Cell uptake for **SGC707**. The intracellular **SGC707** concentrations in RS4;11 and MOLM13 cells were quantified after treatment with 10 μM of **SGC707** for 6 h. Error bars represent mean ± SD from n = 3 technical replicates. (E) **SGC707** stability in RS4;11 cells. **SGC707** concentrations in RS4;11 cells and medium at different time points were quantified after treatment of RS4;11 cells with 1 μM of **SGC707**. The concentration was quantified using an LC-MS/MS method. The concentration of **SGC707** at 0 h is shown as 100%

and the concentration at 48 h is 91%. Error bars represent mean ± SD from n = 3 technical replicates. *P* values (B, D) were calculated by two-way analysis of variance. Significance levels are as follows: * $p < 0.05$, ** $p < 0.01$, *** $p < 0.001$, **** $p < 0.0001$; ns indicates non-significant.

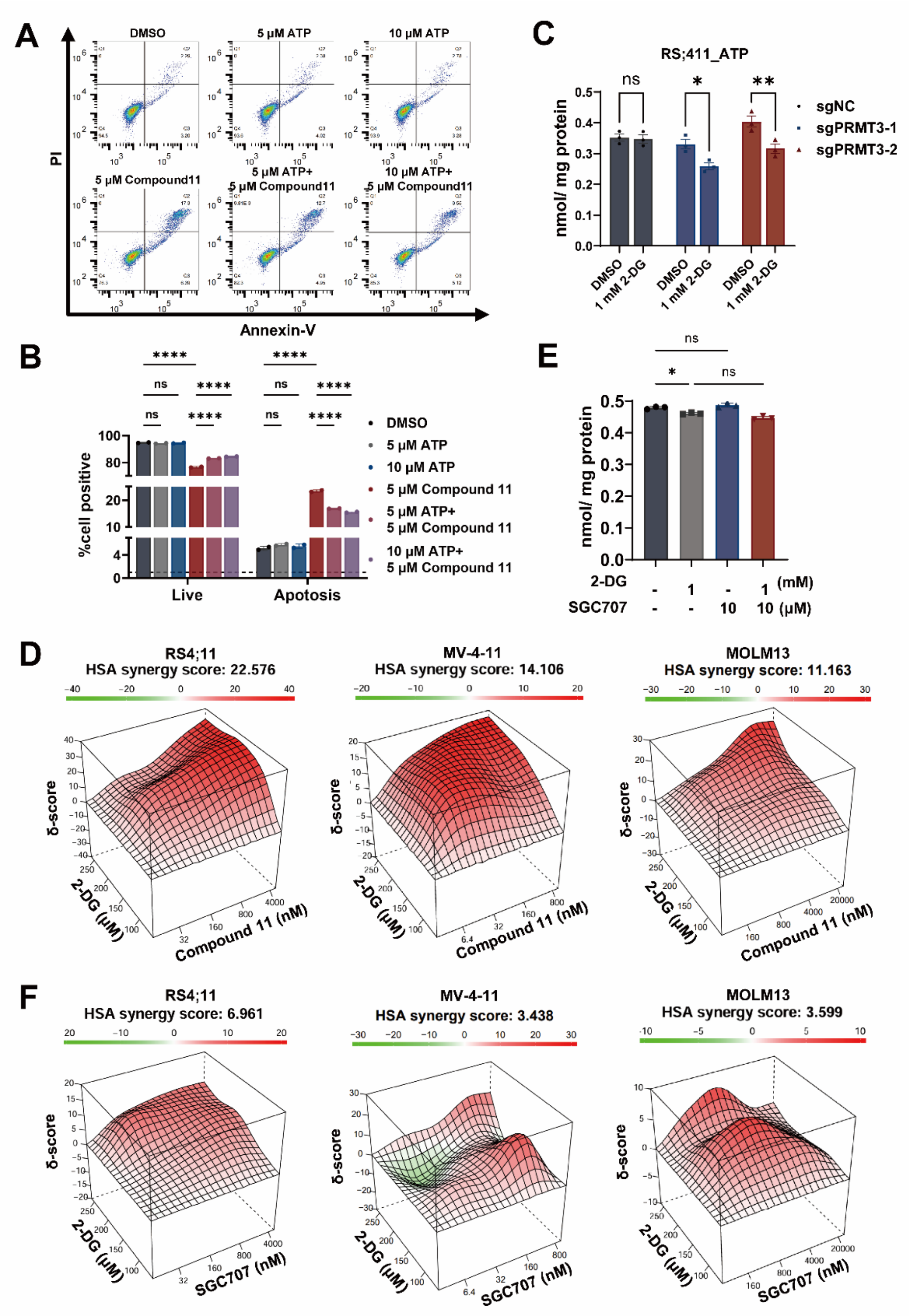

Figure S4. (A、B) Representative flow cytometry analysis of cell apoptosis (A) and quantification (B) of RS4;11 cells treated with compound **11** alone or in combination with ATP for 24 h. Error bars represent mean ± SD from n = 2 technical replicates. (C) Intracellular ATP of RS4;11 WT, sgPRMT3-1 and sgPRMT3-2 cells treated with 1 mM **2-DG** for 24 h. Error bars represent mean ± SD from n = 3 technical replicates. (D) HSA synergy plots for the combination of **11** and **2-DG** in RS;411, MV4-11 and MOLM13 cells (n = 3 technical replicates). (E) Intracellular ATP of RS4;11 treated with **2-DG** or **SGC707** alone and **2-DG** plus **SGC707** at indicated concentration for 24 h. Error bars represent mean ± SD from n = 3 technical replicates. (F) HSA synergy plots for the combination of **SGC707** and **2-DG** in RS4;11, MV-4-11 and MOLM13 cells (n = 3 technical replicates). *P* values (B, C, E) were calculated by two-way analysis of variance. Significance levels are as follows: * p < 0.05, ** p < 0.01, *** p < 0.001, **** p < 0.0001; ns indicates non-significant.

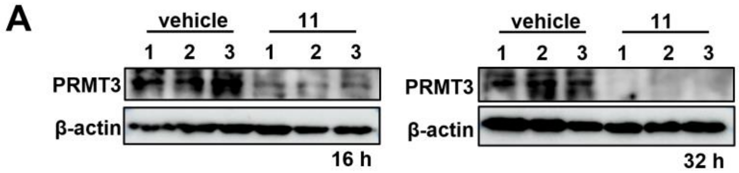

Figure S5. (A) Immunoblot of PRMT3 in plasma at 16 hours and 32 hours after treatment with 100 mg/kg of compound **11** injected intraperitoneally twice daily.

Supplementary Tables

Table S1. PRMT3 degradation of PRMT3 degraders in RS4;11

Compound	Degradation Percentage (%, 24 h)		
	0.1 μM	1 μM	10 μM
1	6.0	9.0	10.3
2	12.7	12.5	7.4
3	3.0	10.8	-1.6
4	1.7	5.6	9.9
5	8.4	4.6	-4.3
6	1.0	7.7	9.4
7	-4.8	4.6	-4.3

8	3.3	6.7	26.2
9	-4.0	1.8	14.2
10	21.9	24.7	-8.7
11	-3.4	42.9	58.4
SGC707	-0.9	2.0	8.0

Table S2. PRMT3 degradation of PRMT3 degraders in MV-4-11

Compound	Degradation Percentage (%, 24 h)		
	0.1 μM	1 μM	10 μM
1	-2.3	1.8	2.3
2	-2.5	0.8	-1.9
3	-1.8	1.0	-0.1
4	-2.0	1.0	-0.4
5	0.7	5.5	9.7
6	7.6	12.7	12.5
7	8.1	11.8	10.3
8	-16.5	8.6	41.4
9	7.2	7.5	23.6
10	-0.1	5.0	34.1
11	15.8	25.8	54.3
SGC707	0.9	-2.0	-3.0

Table S3. Degradation of different proteins by compound **11** in RS4;11 cells

Compound	Concentration/μM	Degradation Percentage (%, 24 h)		
		PRMT3	PRMT5	PRMT6
DMSO	-	0.0	0.0	0.0
Compound 11	1	3.5	0.3	2.4
	2.5	13.7	3.7	-3.9
	5	29.8	6.0	-5.4
	7.5	38.4	18.0	-1.1
	10	44.9	9.2	-4.8

Table S4. PRMT3 degradation of compound **11** in MOLM13 and MOLT4 cells.

Compound	Concentration/μM	Degradation Percentage (%, 24 h)	
		MOLM13	MOLT4
DMSO	-	0.0	0.0
Compound 11	1	4.1	2.1
	2.5	25.3	8.5

	5	35.9	31.7
	7.5	41.3	43.6
	10	44.4	44.3

Table S5. PRMT3 degradation of different compounds at indicated concentration.

Compound	Concentration	Degradation Percentage (%, 24 h)
Compound 11	0.1	4
	1	-4
	10	45
PRMT3-i	0.1	1
	1	0
	10	8
MDM2-i	0.1	6
	1	6
	10	9

Table S6. The PRMT3 and MDM2 enzyme activity inhibition.

Compound	IC_{50}(μM)	
	PRMT3	MDM2
PRMT3-i	7.9	-
MDM2-i	-	0.8

Table S7. PRMT3 degradation of different compounds at indicated time points.

Compound	Time/h	Degradation Percentage (%, 24h)
DMSO	48	0
20 μM CHX	6	-0.1
	12	-9.8
	24	16.0
	36	69.2
	48	67.0
20 μM CHX+10 μM compound 11	6	3.0
	12	25.6
	24	59.4
	36	69.5
	48	70.1

Table S8. Relative pan-Asymmetric-Di-Methyl Arginine protein level in different cells treated with indicated concentrations of **11** and **SGC707**.

Compound	DMSO	Compound 11					SGC707
(concentration/μM)	-	1	2.5	5	7.5	10	10
RS4;11	100.0	95.3	73.0	71.2	49.4	49.0	90.2
MV-4-11	100.0	101.1	101.7	101.8	79.9	79.2	74.2
MOLM13	100.0	96.0	95.9	94.8	91.3	79.2	70.9
Mino	100.0	99.7	86.4	87.4	87.3	82.6	99.6

Table S9. Relative band intensity (%) of different proteins in Fig 4D.

Compound	Concentration/μM	Relative band intensity(%)	
		Bax	CHOP
DMSO	-	100.0	100.0
Compound 11	1	107.2	101.2
	2.5	164.4	104.0
	5	219.7	116.0
	7.5	212.6	123.1
	10	242.1	138.3

Table S10. Relative band intensity(%) of different proteins in Fig 4E.

Compound	Concentration/μM	Relative band intensity(%)			
		PARP	Cle-PARP	p-H2AX	Cle-caspase3
DMSO	-	100.0	100.0	100.0	100.0
Compound 11	1	102.3	151.3	133.8	133.0
	2.5	91.1	161.3	190.7	219.0
	5	73.1	162.1	226.9	316.6
	7.5	60.7	163.5	230.4	332.2
	10	56.3	170.4	265.3	381.2

Table S11. Relative band intensity(%) of different proteins in Fig 5F.

concentration		Relative band intensity(%)	
Compound 11 (μM)	2-DG (mM)	PARP	Cle-PARP
-	-	100.0	100.0
-	0.5	100.9	117.3
-	1	103.4	154.6
2.5	-	105.7	872.8
5	-	99.3	1104.0
2.5	0.5	109.7	1252.3

5	0.5	91.5	1470.2
2.5	1	98.9	1298.1
5	1	88.5	1546.2

Table S12. Pharmacokinetic (PK) Study of compound **11**.[a]

cpd	route (dose)	$T_{1/2}$ (h)	Vd_{ss} (mL/kg)	C_{max} (ng/mL)	AUC_{0-t} (ng·h/mL)	CL (mL/min/kg)	F (%)
**11**	IP (20 mg/kg)	3.4		6907	13781		58.6
**11**	IV (20 mg/kg)	2.58	468		23525	14.1	

[a] Five animals was used for each dose group.

Supplementary Methods

Cell culture

All cells were obtained from the American Type Culture Collection (ATCC) and grown in a humidified incubator held at 37 °C and 5% CO_2. RS4;11, BDCM and Toledo cells were cultured in RPMI 1640 medium (1640, GIBCO) supplemented with 10% (v/v) fetal bovine serum and 0.1% penicillin-streptomycin. Mino and JeKo-1 cells were cultured in RPMI 1640 medium (1640, GIBCO) supplemented with 15% (v/v) fetal bovine serum and 0.1% penicillin-streptomycin. MOLM13 and MOLT4 cells were cultured in RPMI 1640 medium (1640, GIBCO) supplemented with 20% (v/v) fetal bovine serum and 0.1% penicillin-streptomycin. MV-4-11 cells were cultured in Iscove's Modified Dulbecco's Medium (IMDM, GIBCO) supplemented with 10% (v/v) fetal bovine serum and 0.1% penicillin-streptomycin. HL-60 cells were cultured in Iscove's Modified Dulbecco's Medium (IMDM, GIBCO) supplemented with 20% (v/v) fetal bovine serum and 0.1% penicillin-streptomycin. NCI-H358 and HepG2 cells were cultured in Dulbecco's Modified Eagle Medium (DMEM, GIBCO) supplemented with 10% (v/v) fetal bovine serum and 0.1% penicillin-streptomycin.

Western blotting

Cells were treated with various concentrations of compounds for indicated time or various times of

compounds at the indicated concentration. Following the treatment, cells were rinsed once with PBS and were mixed with 1x loading (diluted from premixed 4x Laemmli protein sample buffer solution with PBS, BIO-RAD). After denatured for 10 ~ 15 min at 100 °C, protein markers and samples were loaded on 12% SDS-PAGE gels which were made using standard protocols. Electrophoresis was performed at a constant current of 20 mA per gel for 2 ~ 3 h. Proteins were transferred onto 0.45 μm pore-sized nitrocellulose membranes (Cytiva). After being blocked with 5% [w/v] milk in 1X TBST (25 mM Tris-HCl, pH 7.5, 150 mM NaCl, and 0.1% [v/v] Tween 20) for 1 h at room temperature on a shaking platform, the membranes were incubated with primary antibodies at 4 °C overnight. Upon primary antibodies blotting, the membranes were washed for 10 min by TBST three times and subsequently blotted by secondary antibodies for 1 h at room temperature and wash for 10 min by TBST three times. The results were visualized using BIO-RAD ChemiDoc Touch Imaging System and analyzed with Image Lab software.

The antibodies against PRMT3 (Cat# 72593S, used at 1:1000), BAX(Cat# 2772S, used at 1:1000), PARP (Cat# 9542S, used at 1:1000) , MDM2 (Cat# 86934S, used at 1:1000), Cle-caspase3 (Cat# 9661S, used at 1:1000), Phospho-Histone H2A.X (Cat# 2577S, used at 1:1000) were purchased from Cell Signaling Technology (Massachusetts, USA). The antibodies against PRMT5 (Cat# A2290, used at 1:1000), PRMT6 (Cat# A7814, used at 1:1000), VHL (Cat# A21083, used at 1:1000) , pan-Asymmetric-Di-Methyl Arginine (Cat# A18262, used at 1:1000) were purchased from ABclonal (Wuhan, China). CRBN antibody (Cat# 28494-1-AP, used at 1:1000) and α-Tubulin (Cat# 66031-1-Ig, used at 1:1000), were purchased from Proteintech (Wuhan, China). β-actin antibody (Cat# AM1021B, used at 1:10000) was purchased from Abcepta (Jiangsu, China).

Cell viability assay

The indicated cells were seeded in 96 well plates. MV-4-11, MOLM13, and Mino cells were plated at a density of 8000 cells per well in 80 μL media. RS4;11 cells were plated at a density of 30000 cells per well in 80 μL media. Toledo cells were plated at a density of 3000 cells per well in 80 μL media. Then indicated compounds were added for a final volume of 100 μL. The cells were incubated with compounds for 72 h at 37 °C and 5% CO_2. After treatment,10 μL per well cell counting kit-8 (CCK8) was added and incubated at 37 °C for 1 ~ 2 h. The absorbance of the

tetrazolium salt WST-8 was measured at 450 nm and 650 nm by a microplate reader (SpectraMax 190, Molecular Devices). Cell viability was normalized to the negative control (DMSO). Data were analyzed by GraphPad Prism 8.1.4 software. N = 3 biological replicates were used for each treatment condition.

Cell proliferation analysis

Cells were seeded in 96 well plates at a density of 30000 cells per well in 80 μL media. Then indicated compounds were added for a final volume of 100 μL. Then imaged cells using a phase contrast channel in the IncuCyte S3 platform (Sartorius). A set of phase contrast images were taken every 6 hours from the same area in each well using a 4x objective. IncuCyte S3 image analysis software was set to detect the edges of the cells and to determine their confluence in percentage.

Cell cycle analysis

RS4;11 cells were seeded in 6 well plates at a density of 8×10^5 with 1.8 mL media per well and indicated compounds were added for a final volume of 2 mL. After treatment, cells were washed with cold PBS. Cell pellets were then fixed with 1 mL cold 70% ethanol in PBS at 4 °C overnight. Next, the cell pellets were washed by adding 1 mL PBS, and the cells were resuspended in cell cycle staining solution followed by 10 min incubation at room temperature in the dark. Cell cycle data were acquired by flow cytometry on a Beckman flow cytometer using the Cytexpert software. Data was analyzed using FlowJo. N = 3 biological replicates were used for each treatment condition.

Apoptosis assay

RS4;11 cells were seeded in 6 well plates at a density of 8×10^5 with 1.8 mL media per well and indicated compounds were added for a final volume of 2 mL. After treatment, cells were washed with cold PBS twice. Cells were resuspended in Annexin V binding buffer with FITC annexin V and incubated at room temperature protected from light for 15 min according to the manufacturer's instructions (YEASEN). Analysis was performed on a Beckman flow cytometer using the Cytexpert software within 1 h and processed on FlowJo.

Mitochondrial Membrane Potential Measurement

RS4;11 cells were seeded in 6 well plates at a density of 8×10^5 with 1.8 mL media per well and indicated compounds were added for a final volume of 2 mL. After 24 h treatment, the cells were incubated with JC-1 at 37 °C for 20 min and washed twice with PBS according to the manufacturer's instructions of the JC-1 assay kit. Analysis was performed on a Beckman flow cytometer using the Cytexpert software within 1 h and processed on FlowJo.

Cell uptake and stability

The RS4;11 and MOLM13 cells were seeded in 6 well plates at a density of 1.3×10^6 with 1.8 mL media per well and indicated compounds were added for a final volume of 2 mL. After 6 h treatment, the cells were collected and lysed using methanol/water (8/2, v/v), and the SGC707 concentrations in cells were quantified by using the LC-MS/MS methods.

For testing the stability of SGC707, RS4;11 cells were treated with 1 μM of SGC707. At 0, 24, and 48h, both the medium and cells were collected together and methanol/water (8/2, v/v) was added. Then the SGC707 concentrations were quantified by an LC-MS/MS method.

RT-q-PCR

Extracted the co-precipitated RNA by TRIzol™ Reagent and Phenol-chloroform method. Reverse transcription was performed with PrimerScript RT Master Mix (Takara). Real-time quantitative PCR assays were performed using AceQ® Universal SYBR qPCR Master Mix (Vazyme) and Agilent Technologies Mx3005P sequence detection system. To ensure that equal amounts of cDNA were added to the PCRs, internal standards were using the ACTB and VCL. An evaluation of the relative mRNA levels among the treatment groups was carried out using the 2-ΔΔCT method. Data are presented as relative fold changes compared to the control group. Table S13 includes detailed information about the sequence of the used primers.

Table S13. The primers used in present study

Genes	Forward primer (5'- 3')	Reverse primer (5'- 3')
PRMT3	GTACCCTTCATACCCCAATGG	GACGAGCAGGTTCTGACATCT
HSPA5	CATCACGCCGTCCTATGTCG	CGTCAAAGACCGTGTTCTCG
ATF5	TGGCTCGTAGACTATGGGAAA	ATCAACTCGCTCAGTCATCCA

ATF3	CCTCTGCGCTGGAATCAGTC	TTCTTTCTCGTCGCCTCTTTTT
CEBPB	CTTCAGCCCGTACCTGGAG	GGAGAGGAAGTCGTGGTGC
NDUFS6	TTCGGTTTGTAGGTCGTCAGA	CCATCGCACGCTATCACCC
NDUFAB1	ATGGCGTCTCGTGTCCTTTC	AACCTGCGCGAGCACTAAG
NDUFA1	ATGTGGTTCGAGATTCTCCCC	CCTGTGGATGTACGCAGTAGC
COX7B	CTTGGTCAAAAGCGCACTAAATC	AAAATCAGGTGTACGTTTCTGGT
BAX	CCCGAGAGGTCTTTTTCCGAG	CCAGCCCATGATGGTTCTGAT
ERN1	CACAGTGACGCTTCCTGAAAC	GCCATCATTAGGATCTGGGAGA
BRSK2	AAAGCTGCACGACGTTTATGA	TGCGATGCGGATGTTGTTCT
CDC20	GCACAGTTCGCGTTCGAGA	CTGGATTTGCCAGGAGTTCGG
PLK1	AAAGAGATCCCGGAGGTCCTA	GGCTGCGGTGAATGGATATTTC
MELK	TCTCCCAGTAGCATTCTGCTT	TGATCCAGGGATGGTTCAATAGA
DDIT3	GGAAACAGAGTGGTCATTCCC	CTGCTTGAGCCGTTCATTCTC
BIRC5	AGGACCACCGCATCTCTACAT	AAGTCTGGCTCGTTCTCAGTG
NME1	AAGGAGATCGGCTTGTGGTTT	CTGAGCACAGCTCGTGTAATC
AURKB	CAGTGGGACACCCGACATC	GTACACGTTTCCAAACTTGCC
AURKA	GAGGTCCAAAACGTGTTCTCG	ACAGGATGAGGTACACTGGTTG
CDC25A	GTGAAGGCGCTATTTGGCG	TGGTTGCTCATAATCACTGCC
PTTG1	ACCCGTGTGGTTGCTAAGG	ACGTGGTGTTGAAACTTGAGAT
SPAG5	TTGAGGCCCGTTTAGATACCA	GCTTTCCTTGGAGCAATGTAGTT
LDHA	ATGGCAACTCTAAAGGATCAGC	CCAACCCCAACAACTGTAATCT
TIMM10	TCCAAGGGCGAGTCTGTGT	AACTTTTTGCCCATCCGCTCA
VHL	CTCGTCCGGGTTGGAAAAGAG	AGTAAGGGTCTGACTGAAGCAT
ACTB	CATGTACGTTGCTATCCAGGC	CTCCTTAATGTCACGCACGAT

ATP measurements

RS4;11 cells were seeded in 6-well plates at a density of 1×10^6 with 1.8 mL media per well and the indicated compounds were added to a final volume of 2 mL. Cells were collected and washed with cold PBS after 24 h treatment. Intracellular ATP levels were determined using the luciferase-based assay (Beyotime, S0027) based on the manufacturer's instructions. Luminescence was measured

using a luminometer (En Vision).

PRMT3 methyltransferase activity assay

Dilute the compound to be tested from 50 μM by 7 doses with DMSO and the compounds were diluted by 4 folds. The compounds are incubated for 60 min at 22°C with about 300 ng human PRMT3 enzyme, 350 nM [3H]SAM and 250 nM Histone H4 full length in a buffer containing 45 mM Tris-HCl (pH 9), 45 mM NaCl, 4.5 mM $MgCl_2$ and 3.6 mM DTT. For control basal measurements, the enzyme is omitted from the reaction mixture. Following incubation, the reaction is stopped by adding 33 mM citric acid and the samples are filtered rapidly under vacuum through glass fiber filters (GF/B, Packard) presoaked with 33 mM citric acid and rinsed several times with ice-cold 33 mM citric acid using a 96-sample cell harvester (Unifilter, Packard). The filters are dried then counted for radioactivity in a scintillation counter (Topcount,Packard) using a scintillation cocktail (Microscint 0, Packard). The results are expressed as a percent inhibition of the control enzyme activity. The standard reference compound is SAH, which is tested in each experiment at several concentrations to obtain a competition curve from which its IC_{50} is calculated.

FP assays for MDM2/p53 protein-protein interactions

MDM2 (1-118) protein were expressed in BL21 (DE3) strain of *E. coli* and purified through nickel affinity chromatography and subsequently gel filtration chromatography. The FAM labeled PDI peptide (FAM-PDI)[1] was used as a tracer for MDM2 binding assay, and the K_d values tested for MDM2 are 0.7 nM in the saturation experiments.

In the competitive binding assay, the fluorescence polarization values were measured using the Spark™ 10M plate reader in 96-well, black, round-bottom plates. Two components were added sequentially: (1) 4 μL of the serial diluted testing compounds in DMSO; (2) 96 μL of the mixture of the assay buffer, the tracer FAM-PDI, and corresponding protein. The components of the assay buffer included 100 mM potassium phosphate (pH 7.5) + 100 ug/mL Bovine-γ-globulin + 0.01% TritonX-100 for MDM2. The final concentration of the tracer in each well is 2 nM in MDM2 system. The final concentration of individual testing protein of interest is 20 nM for MDM2. All the components were mixed in each well of the plates, which was incubated at room temperature for 1

h with gentle shaking. The polarization values in millipolarization units (mP) were measured at an excitation wavelength of 485 nm and an emission wavelength of 530 nm. The IC_{50} values were calculated with Graphpad. The K_i values were calculated based on the equation $K_i = IC_{50}/(1+[L]/K_d)$ following reported calculation methods.

CRISPR-mediated PRMT3 knockout

To generate PRMT3 knockout cells, CRISPR-Cas9 genome editing was performed. sgRNA targeting PRMT3 was cloned into plasmid lentiGuide-Puro (https://www.addgene.org/52963/) vector. The lentivirus was produced by using a two-plasmid packaging system according to the previous report.[1] Cells were infected with lentivirus, and selected with puromycin (1 μg/mL) for a week. Analysis of PRMT3 knockout with immunoblotting revealed that guide sequences showed in table S14 provided the strongest consistent decrease in PRMT3 levels among assayed sgRNAs. These guides were designated sgPRMT3-1 and sg PRMT3-2, respectively.

Table S14. Sequences of RNA Oligonucleotides

Name	Sense strand/sense primer (5’- 3’)	Antisense strand/antisense primer (5’- 3’)
sgPRMT3-1	GGCTGATAGTGCAAATGTCA	TGACATTTGCACTATCAGCC
sgPRMT3-2	GAATTCATGTACTCAACTGT	ACAGTTGAGTACATGAATTC
sgNC	GACCGGGGCGAGGAGCTGTTCACCG	CGGTGAACAGCTCCTCGCCCCGGTC

Pharmacokinetic and pharmacodynamics study in mouse

Female BALB/c mice (19-25g) employed in PK study were fasted for 4 h before administration and remained fasting for 2 h. The animal room environment was controlled (target condition: temperature 18-29 °C, relative humidity 30 to 70%). Temperature and relative humidity were monitored daily. Female BALB/c mice (19-25g) (n = 5) were administered intraperitoneally or intravenously with compound 11 which was formulated in DMSO, Solutol HS15 and saline (5:5:90, v/v/v) to give a final concentration of 2 mg/mL at a single 20 mg/kg dose. Blood samples (approximately 60 μL) were collected at 0.083, 0.25, 0.5, 1, 2, 4, 6 and 24 h. At each time point, blood samples were collected from five mice. Immediately after collection, plasma was harvested

by centrifugation and stored at -80°C until analysis. The samples were analyzed by LC-MS/MS (Water XEVO TQ-S). The data acquisition (including C_{max}, $T_{1/2}$, T_{max} and AUC) and control system were created using WinNonLin 8.0.

For pharmacodynamics study, six female BALB/c mice were randomly divided (n=3) and treated with 100 mg/kg of compound 11 which was formulated in DMSO, Solutol HS15 and saline (5:5:90, v/v/v) to give a final concentration of 10 mg/mL or vehicle intraperitoneally twice a day. The blood was collected and lysed with RIPA lysate for western blot analysis.

Chemical Synthesis

Methods and materials. All the reagents and solvents were purchased from commercially available companies. Unless otherwise noted, commercial solvents and reagents were used without further purification. Silica gel 60H (200 − 300 mesh) manufactured by Qingdao Haiyang Chemical Group Co. (China) was used for general chromatography. The final compounds were all purified by C18 reversed-phase preparative high-performance liquid chromatography (HPLC) column with H_2O and CH_3CN as eluents. The purity of all of the final compounds was confirmed to be > 95% by HPLC analysis with Agilent ZORBAX SB-C18 reversed-phase column (250mm × 4.60mm, 5 μm). Conditions were as follows: flow rate = 1.0 mL/min, CH3CN/H2O eluent (containing 0.1% trifluoroacetic acid); gradient, 5% CH3CN to 100% CH3CN; 25 min; monitored by UV absorption at 254 nm. Reactions were monitored by thin-layer chromatography (TLC) or liquid chromatography−mass spectrometry (LC−MS). LC−MS was performed by Waters ultraperformance liquid chromatography (UPLC) H-Class with ACQUITY UPLC BEH C18 reversed-phase column (2.1 mm × 50 mm, 1.7 μm, flow rate = 0.5 mL/min). Mass analysis was performed by the Waters SQD2 single quadrupole mass detection system. ^{1}H NMR and ^{13}C NMR spectra were determined with BRUKER AVANCE II 400 M or BRUKER AVANCE III 600 M NMR spectrometer. High-resolution electrospray ionization (ESI) mass analysis was performed by an Agilent 1290−6545 UHPLC-QTOF high-resolution mass spectrometer (MS).

Chemical synthesis of key intermediate 15 and PRMT3-i.

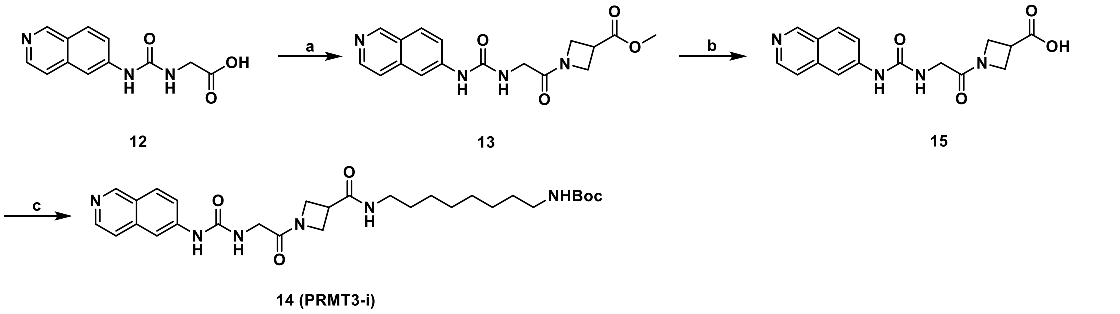

Scheme S1. **Reaction conditions:** (a) methyl azetidine-3-carboxylate (hydrochloride), HATU, DIEA, DMF, rt., 4 h; (b) 2M NaOH, THF / DMF, rt., 3 h; (c) tert-Butyl(8-aminooctyl) carbamate, HATU, DIEA, DMF, rt., 4 h. Compound **12** was synthesized according to published procedures.[2]

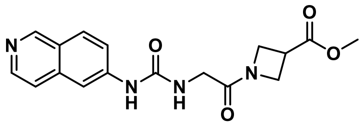

*methyl 1-((isoquinolin-6-ylcarbamoyl)glycyl)azetidine-3-carboxylate (**13**).* To a solution of **12** (2 g, 8.2 mmol) in DMF was added methyl azetidine-3-carboxylate (hydrochloride) (1.244 g, 8.2 mmol), HATU (4.032 g, 10.6 mmol), and DIEA (7.1 mL, 40.8 mmol). The resulting mixture was stirred at room temperature for 1 h. After the reaction finished, the mixture was directly purified by flash column chromatography (0 - 15% MeOH in DCM) to yield **13** (2.12 g, 75% yield).

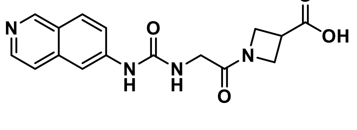

*1-((isoquinolin-6-ylcarbamoyl)glycyl)azetidine-3-carboxylic acid (**intermediate 15**).* To the solution of **13** (2.12 g, 6.2 mmol) in THF / DMF (THF : DMF = 30 mL : 10 mL), 2M NaOH (9.3 mL, 18.6 mmol) was added. And the mixture was stirred at room temperature for 1 h until the reaction was completed. Then the reaction was quenched by 1M HCl. The mixture was purified by reverse phase flash column chromatography (0 - 40% MeOH in water) to yield the desired intermediate **15** (1.78 g, 88% yield). ^{1}H NMR (600 MHz, DMSO-d_6) δ 10.02 (s, 1H), 9.50 (s, 1H), 8.45 (d, J = 6.5 Hz, 1H), 8.36 (d, J = 2.1 Hz, 1H), 8.31 (d, J = 9.0 Hz, 1H), 8.14 (d, J = 6.5 Hz, 1H),

7.78 (dd, J = 9.0, 2.1 Hz, 1H), 6.97 (t, J = 5.3 Hz, 1H), 4.37 (t, J = 8.8 Hz, 1H), 4.26 (dd, J = 8.5, 5.9 Hz, 1H), 4.07 (t, J = 9.3 Hz, 1H), 3.93 (dd, J = 9.5, 5.9 Hz, 1H), 3.80 (d, J = 5.1 Hz, 2H), 3.47 (tt, J = 9.2, 5.9 Hz, 1H). ^{13}C NMR (151 MHz, DMSO-d_6) δ 173.7, 168.7, 154.5, 146.7, 146.2, 139.6, 133.5, 131.3, 122.8, 122.6, 122.5, 109.8, 51.9, 50.5, 32.1.

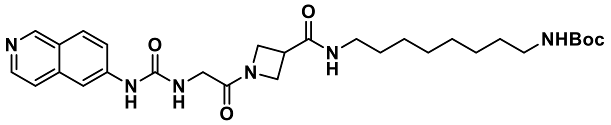

tert-butyl(8-(1-((isoquinolin-6-ylcarbamoyl)glycyl)azetidine-3-carboxamido)octyl)carba mate ***(14, PRMT3-i)***. To a solution of **15** (50 mg, 0.15 mmol) in DMF was added tert-Butyl(8-aminooctyl)carbamate (38 mg, 0.15 mmol), HATU (75 mg, 0.2 mmol), and DIEA (0.15 mL, 0.75 mmol). The resulting mixture was stirred at room temperature for 1 h. After the reaction finished, the mixture was directly purified by C18 reverse phase preparative high-performance liquid chromatography to give the **14** (**PRMT3-*i***) as a white solid (40 mg, 47% yield). ^{1}H NMR (400 MHz, Methanol-d_4) δ 9.0 (s, 1H), 8.2 (d, J = 5.9 Hz, 1H), 8.0 (d, J = 2.0 Hz, 1H), 7.9 (d, J = 8.9 Hz, 1H), 7.5 (d, J = 5.9 Hz, 1H), 7.5 (dd, J = 8.9, 2.0 Hz, 1H), 4.4 (t, J = 8.7 Hz, 1H), 4.4 (dd, J = 8.7, 5.9 Hz, 1H), 4.2 (t, J = 9.3 Hz, 1H), 4.1 (dd, J = 9.8, 5.9 Hz, 1H), 4.0 – 3.8 (m, 2H), 3.5 (tt, J = 8.8, 5.8 Hz, 1H), 3.2 (t, J = 7.1 Hz, 2H), 3.0 (t, J = 7.1 Hz, 2H), 1.5 – 1.4 (m, 15H), 1.3 – 1.2 (m, 10H).

Chemical synthesis of PRMT3 PROTACs 1-11.

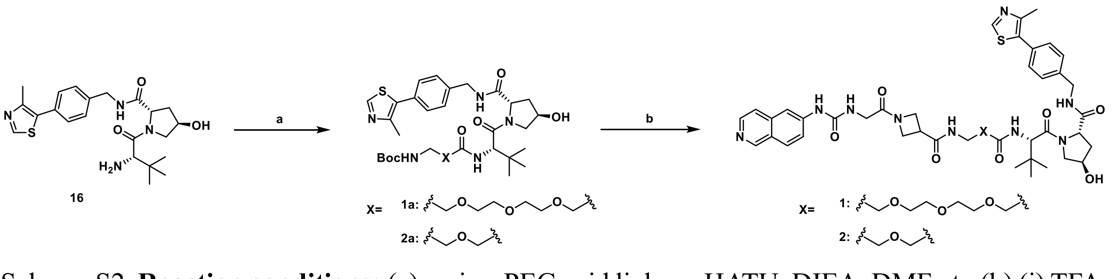

Scheme S2. **Reaction conditions:** (a) amino-PEG acid linkers, HATU, DIEA, DMF, rt.; (b) (i) TFA, DCM, rt., 1 h; (ii) **15**, HATU, DIEA, DMF, rt., 3 h. Compound **16** was synthesized according to published procedures.[3]

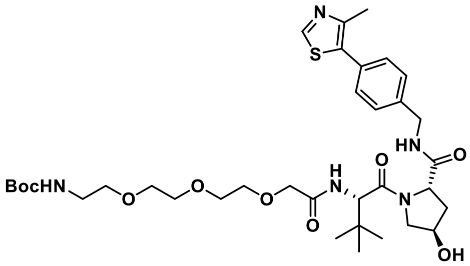

*tert-butyl((S)-13-((2S,4R)-4-hydroxy-2-((4-(4-methylthiazol-5-yl)benzyl)carbamoyl)pyrroli dine-1-carbonyl)-14,14-dimethyl-11-oxo-3,6,9-trioxa-12-azapentadecyl)carbamate (**1a**)*. To a solution of **16** (200 mg, 0.47 mmol) and tert-butyl 2,2-dimethyl-4-oxo-3,8,11,14-tetraoxa-5-azahexadecan-16-oic acid (116 mg, 0.47 mmol) in DMF (3 mL), was added DIEA (0.33 mL, 2.3 mmol) followed by HATU (186 mg, 0.6 mmol). The mixture was stirred for 3 h remaining room temperature. After finishing, **1a** was gained via EA extraction, concentration, and silica gel purification (0 - 10% MeOH in DCM, 248 mg, 72% yield). LC-MS (ESI) calculated for $C_{35}H_{53}N_5O_9S$ $[M + Na]^+ = 742.36$, found 742.20.

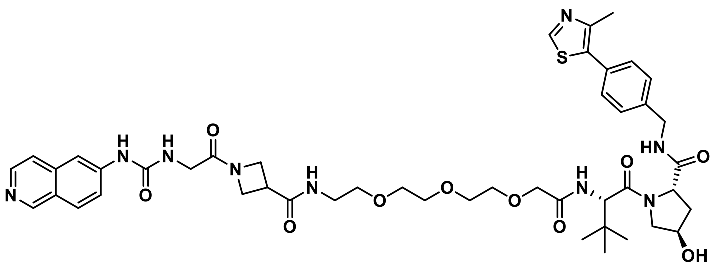

*(2S,4R)-4-hydroxy-1-((S)-2-(1-(1-((isoquinolin-6-ylcarbamoyl)glycyl)azetidin-3-yl)-1-oxo-5,8,11-trioxa-2-azatridecan-13-amido)-3,3-dimethylbutanoyl)-N-(4-(4-methylthiazol-5-yl)benzyl)pyroli-dine-2-carboxamide (**1**).* **1a** (43 mg, 0.06 mmol) was dissolved in DCM : TFA (5 mL : 1 mL) and the mixture was stirred at room temperature for 0.5 h. The reaction was then concentrated under reduced pressure to give deprotection intermediate which was used for the next step without purification. To a solution of deprotection intermediate and **15** (20 mg, 0.06 mmol) in DMF (3 mL), DIEA (52 μL, 0.3 mmol) was added followed by HATU (29 mg, 0.08 mmol). The mixture was stirred at room temperature for 3 h. After finishing, the mixture was purified by C18 reverse phase preparative high-performance liquid chromatography to give the final compound **1** as a white solid (23 mg, 36% yield). HRMS $(M + H)^+$ calcd for $C_{46}H_{60}N_9O_{10}S^+$ 930.4178, found 930.4176. ^1H NMR (400 MHz, DMSO-d_6) δ 10.08 (d, J = 7.6 Hz, 1H), 9.57 (s, 1H), 8.99 (s, 1H), 8.60 (t, J = 6.0 Hz,

1H), 8.46 (d, J = 6.6 Hz, 1H), 8.41 (d, J = 2.3 Hz, 1H), 8.37 (d, J = 9.1 Hz, 1H), 8.24 (dd, J = 6.7, 2.5 Hz, 1H), 8.10 (dt, J = 13.8, 5.5 Hz, 1H), 7.83 (dd, J = 9.1, 2.0 Hz, 1H), 7.46 – 7.35 (m, 5H), 6.96 (dt, J = 11.4, 5.3 Hz, 1H), 4.57 (dd, J = 9.5, 1.9 Hz, 1H), 4.49 – 4.13 (m, 6H), 4.01 – 3.85 (m, 3H), 3.80 (d, J = 4.7 Hz, 2H), 3.70 – 3.48 (m, 11H), 3.41 (td, J = 5.9, 3.1 Hz, 3H), 3.24 (p, J = 5.7 Hz, 2H), 2.43 (d, J = 2.0 Hz, 3H), 2.11 – 2.02 (m, 1H), 1.90 (ddd, J = 13.1, 8.8, 4.5 Hz, 1H), 0.94 (d, J = 1.8 Hz, 9H). ^{13}C NMR (151 MHz, DMSO-d_6) δ 171.9, 171.4, 169.2, 169.1, 168.7, 168.5, 154.5, 154.4, 151.6, 147.8, 147.7, 145.2, 140.3, 139.5, 131.9, 131.6, 131.3, 129.7, 128.8, 127.5, 123.3, 123.2, 122.2, 109.7, 70.5, 69.8, 69.7, 69.6, 69.1, 69.0, 68.9, 58.8, 56.7, 55.8, 52.0, 50.5, 42.5, 41.7, 38.8, 38.7, 38.0, 35.8, 32.5, 26.2, 15.9.

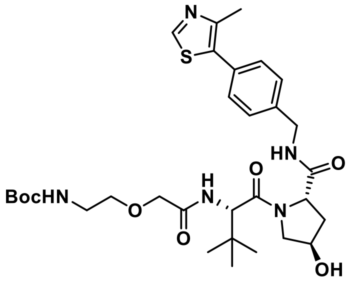

*tert-butyl (2-(2-(((S)-1-((2S,4R)-4-hydroxy-2-((4-(4-methylthiazol-5-yl)benzyl)carbamoyl)pyrolidin -1-yl)-3,3-dimethyl-1-oxobutan-2-yl)amino)-2-oxoethoxy)ethyl)carbamate (**2a**).* To a solution of **16** (200 mg, 0.1 mmol) and 2-(2-((tert-butoxycarbonyl)amino)ethoxy)acetic acid (83 mg, 0.47 mmol) in DMF (3 mL), was added DIEA (0.33 mL, 2.3 mmol) followed by HATU (186 mg, 0.6 mmol). The mixture was stirred for 3 h remaining room temperature. After finishing, **2a** was gained via EA extraction, concentration, and silica gel purification (0 - 10% MeOH in DCM, 220 mg, 75% yield). LC-MS (ESI) calculated for $C_{31}H_{45}N_5O_7S$ [M + Na]$^+$ = 654.30, found 654.15.

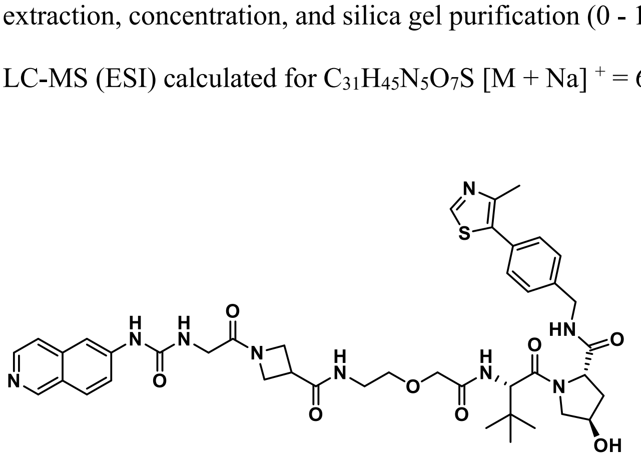

(2S,4R)-4-hydroxy-1-((S)-2-(2-(2-(1-((isoquinolin-6-ylcarbamoyl)glycyl)azetidine-3-carboxamido) ethoxy)acetamido)-3,3-dimethylbutanoyl)-N-(4-(4-methylthiazol-5-yl)benzyl)pyrrolidine-2-carbox-

*amide (2).*Compound **2** was obtained from **2a** using the same method as **1** synthesized, white solid (25 mg, 38% yield). HRMS $(M + H)^+$ calcd for $C_{42}H_{52}N_9O_8S^+$ 842.3654, found 842.3652. ^1H NMR (600 MHz, DMSO-d_6) δ 10.06 (d, J = 23.5 Hz, 1H), 9.57 (s, 1H), 9.00 (d, J = 7.4 Hz, 1H), 8.60 (dd, J = 7.1, 4.2 Hz, 1H), 8.45 (d, J = 6.6 Hz, 1H), 8.43 – 8.40 (m, 1H), 8.36 (d, J = 9.0 Hz, 1H), 8.22 (dt, J = 8.6, 5.5 Hz, 2H), 7.82 (dd, J = 9.1, 2.0 Hz, 1H), 7.50 – 7.43 (m, 1H), 7.42 – 7.34 (m, 4H), 6.95 (dt, J = 11.6, 5.3 Hz, 1H), 4.57 (dd, J = 9.6, 3.5 Hz, 1H), 4.45 (td, J = 7.4, 6.0, 2.2 Hz, 1H), 4.42 – 4.34 (m, 2H), 4.31 – 4.17 (m, 2H), 4.02 – 3.95 (m, 2H), 3.92 (td, J = 10.0, 9.3, 4.0 Hz, 1H), 3.83 (d, J = 4.5 Hz, 1H), 3.78 (d, J = 4.3 Hz, 1H), 3.69 – 3.60 (m, 2H), 3.53 (tt, J = 13.6, 6.3 Hz, 2H), 3.42 (ttt, J = 8.9, 5.8, 2.6 Hz, 1H), 3.31 (dqq, J = 19.6, 11.7, 5.7 Hz, 2H), 2.43 (d, J = 2.7 Hz, 3H), 2.10 – 2.04 (m, 1H), 1.91 (ddd, J = 12.7, 7.1, 3.1 Hz, 1H), 0.94 (s, 9H). ^{13}C NMR (151 MHz, DMSO-d_6) δ 171.9, 171.5, 168.7, 168.5, 154.5, 154.4, 151.7, 147.8, 147.7, 147.6, 145.2, 140.3, 139.6, 132.0, 131.6, 131.4, 129.7, 128.8, 127.6, 123.3, 123.2, 122.2, 109.8, 69.7, 69.5, 69.0, 58.9, 56.7, 55.9, 52.1, 50.6, 42.6, 41.8, 38.7, 38.6, 38.0, 35.8, 32.6, 26.3, 15.9.

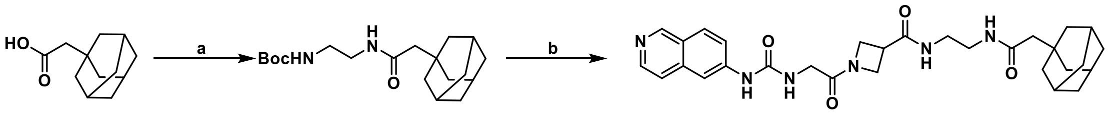

3a 3

Scheme S3. **Reaction conditions:** (a) N-Boc-Ethylenediamine, DIEA, HATU, DMF, rt., 3 h; (b) (i) TFA, DCM, rt., 1 h; (ii) **15**, DIEA, HATU, DMF, rt., 3 h.

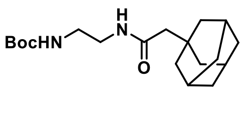

*tert-butyl(2-(2-(adamantan-1-yl)acetamido)ethyl)carbamate (**3a**).* To a solution of 1-adamantaneacetic acid (150 mg, 0.8 mmol) and N-Boc-Ethylenediamine (136 mg, 0.88 mmol) in DMF (3 mL), was added DIEA (0.7 mL, 4 mmol) followed by HATU (382 mg, 1 mmol). The mixture was stirred for 3 h remaining room temperature. After finishing, **3a** was gained via EA extraction, concentration, and silica gel purification (0 - 8% MeOH in DCM, 280 mg, 89% yield). LC-MS (ESI) calculated for $C_{19}H_{32}N_2O_3$ $[M + H]^+$ = 337.24, found 337.03.

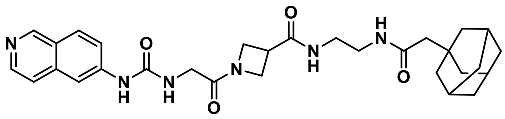

N-(2-(2-(adamantan-1-yl)acetamido)ethyl)-1-((isoquinolin-6-ylcarbamoyl)glycyl)azetidine-3-carboxamide (3). **3a** (50 mg, 0.15 mmol) was dissolved in DCM : TFA (5 mL : 1 mL) and the mixture was stirred at room temperature for 0.5 h. The reaction was then concentrated under reduced pressure to give deprotection intermediate which was used for the next step without purification. To a solution of deprotection intermediate and **15** (49 mg, 0.15 mmol) in DMF (3 mL), DIEA (0.13 mL, 0.75 mmol) was added followed by HATU (73 mg, 0.2 mmol). The mixture was stirred at room temperature for 3 h. After finishing, the mixture was purified by C18 reverse phase preparative high-performance liquid chromatography to give the final compound **3** as a white solid (48 mg, 59% yield). HRMS $(M + H)^+$ calcd for $C_{30}H_{39}N_6O_4^+$ 547.3027, found 547.303. ^{1}H NMR (400 MHz, DMSO-d_6) δ 10.10 (s, 1H), 9.57 (s, 1H), 8.46 (d, J = 6.6 Hz, 1H), 8.40 (d, J = 2.0 Hz, 1H), 8.37 (d, J = 9.1 Hz, 1H), 8.23 (d, J = 6.7 Hz, 1H), 8.11 – 8.06 (m, 1H), 7.81 (dd, J = 9.1, 2.1 Hz, 1H), 7.78 – 7.74 (m, 1H), 6.99 (t, J = 5.2 Hz, 1H), 4.29 (t, J = 8.6 Hz, 1H), 4.19 (dd, J = 8.3, 5.8 Hz, 1H), 3.99 (t, J = 9.1 Hz, 1H), 3.91 (dd, J = 9.3, 5.9 Hz, 1H), 3.79 (d, J = 5.1 Hz, 2H), 3.34 (tt, J = 8.9, 5.9 Hz, 1H), 3.11 (q, J = 5.6 Hz, 4H), 1.92 – 1.85 (m, 3H), 1.80 (s, 2H), 1.66 – 1.51 (m, 12H). ^{13}C NMR (126 MHz, DMSO-d_6) δ 171.3, 170.2, 168.4, 154.4, 147.5, 145.3, 140.1, 131.8, 123.2, 123.1, 122.2, 109.7, 51.9, 50.5, 50.1, 42.1, 38.7, 38.0, 36.4, 32.6, 32.1, 28.0.

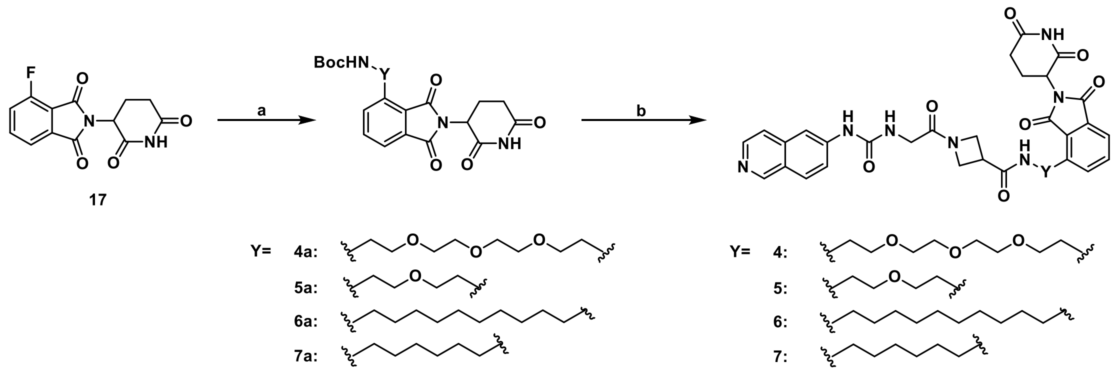

Scheme S4. **Reaction conditions:** (a) diamine linkers, DIEA, DMAc, 90 °C, overnight; (b) (i) TFA, DCM, rt., 1 h; (ii) **15**, DIEA, HATU, DMF, rt., 3 h.

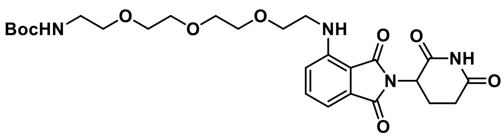

*tert-butyl(2-(2-(2-(2-((2-(2,6-dioxopiperidin-3-yl)-1,3-dioxoisoindolin-4-yl)amino)ethoxy) ethoxy)ethoxy)ethyl)carbamate (**4a**)*. To a solution of **17** (800 mg, 2.9 mmol) and tert-butyl (2-(2-(2-(2-aminoethoxy)ethoxy)ethoxy)ethyl)carbamate (890 mg, 3 mmol) in DMAc (20 mL), was added DIEA (1.51 mL, 8.7 mmol). The mixture was then stirred at 90 °C overnight. After finishing, **4a** was gained via EA extraction, concentration, and silica gel purification (0 - 10% MeOH in DCM, 797 mg, 50% yield). LC-MS (ESI) calculated for $C_{26}H_{36}N_4O_9$ $[M + Na]^+$ = 571.25, found 571.18.

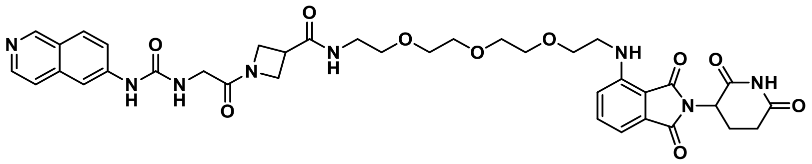

N-(2-(2-(2-(2-((2-(2,6-dioxopiperidin-3-yl)-1,3-dioxoisoindolin-4-yl)amino)ethoxy)ethoxy)ethoxy) ethyl)-1-((isoquinolin-6-ylcarbamoyl)glycyl)azetidine-3-carboxamide (4). **4a** (50 mg, 0.09 mmol) was dissolved in DCM : TFA (5 mL : 1 mL) and the mixture was stirred at room temperature for 0.5 h. The reaction was then concentrated under reduced pressure to give deprotection intermediate which was used for the next step without purification. To a solution of deprotection intermediate and **15** (30 mg, 0.09 mmol) in DMF (3 mL), DIEA (0.08 mL, 0.45 mmol) was added followed by HATU (45 mg, 0.12 mmol). The mixture was stirred at room temperature for 3 h. After finishing, the mixture was purified by C18 reverse phase preparative high-performance liquid chromatography to give the final compound **4** as a yellow solid (23 mg, 33% yield). HRMS $(M + H)^+$ calcd for $C_{37}H_{43}N_8O_{10}^+$ 759.3097, found 759.3095. ^1H NMR (400 MHz, DMSO-d_6) δ 11.1 (s, 1H), 10.1 (s, 1H), 9.6 (s, 1H), 8.5 (s, 1H), 8.4 (d, J = 2.1 Hz, 1H), 8.4 (d, J = 9.1 Hz, 1H), 8.2 (d, J = 6.3 Hz, 1H), 8.1 (t, J = 5.6 Hz, 1H), 7.8 (dd, J = 9.1, 2.0 Hz, 1H), 7.6 (ddd, J = 8.5, 7.1, 4.2 Hz, 1H), 7.1 (dd, J = 8.6, 5.7 Hz, 1H), 7.0 (dd, J = 7.4, 3.4 Hz, 2H), 6.6 (s, 1H), 5.1 (dd, J = 12.9, 5.3 Hz, 1H), 4.3 (t, J = 8.5 Hz, 1H), 4.2 (dd, J = 8.2, 5.8 Hz, 1H), 4.0 (t, J = 9.0 Hz, 1H), 3.9 (dd, J = 9.2, 5.8 Hz, 1H), 3.8 (d, J = 5.1 Hz, 2H), 3.6 (t, J = 5.3 Hz, 2H), 3.6 – 3.3 (m, 16H), 3.2 (p, J = 5.7 Hz, 2H), 2.0 (pt, J = 9.0, 4.3 Hz, 1H). ^{13}C NMR (126 MHz, DMSO-d_6) δ 172.9, 171.3, 170.1, 169.0, 168.5, 167.3, 154.4, 147.5, 146.4, 145.3,140.1, 136.3, 132.1, 131.9, 131.8, 123.2, 117.5, 110.7, 109.7, 109.3, 69.8, 69.6,

69.0, 68.9, 52.0, 50.5, 48.6, 41.7, 38.8, 32.5, 31.0, 22.2.

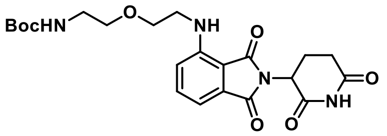

*tert-butyl (2-(2-((2-(2,6-dioxopiperidin-3-yl)-1,3-dioxoisoindolin-4-yl)amino)ethoxy)ethyl)carbamate (**5a**).* To a solution of **17** (1 g, 3.6 mmol) and tert-butyl (2-(2-aminoethoxy)ethyl)carbamate (739 mg, 3.6 mmol) in DMAc (20 mL), was added DIEA (2 mL, 10.8 mmol). The mixture was then stirred at 90 °C overnight. After finishing, **5a** was gained via EA extraction, concentration, and silica gel purification (0 - 10% MeOH in DCM, 1.05 g, 63% yield). LC-MS (ESI) calculated for $C_{22}H_{28}N_4O_7$ [M + Na] $^+$ = 483.20, found 483.02.

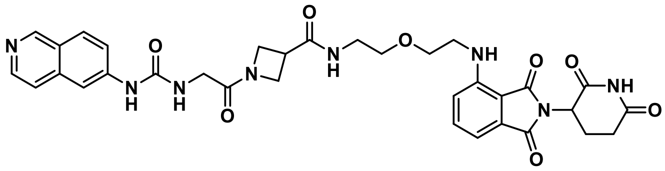

*N-(2-(2-((2-(2,6-dioxopiperidin-3-yl)-1,3-dioxoisoindolin-4-yl)amino)ethoxy)ethyl)-1-((isoquinolin-6-ylcarbamoyl)glycyl)azetidine-3-carboxamide(**5**).* Compound **5** was obtained from **5a** by using the same method as **4** synthesized, yellow solid (35 mg, 48% yield). HRMS $(M + H)^+$ calcd for $C_{33}H_{35}N_8O_8^+$ 671.2572, found 671.2576. ^{1}H NMR (400 MHz, DMSO-d_6) δ 11.1 (s, 1H), 10.2 (s, 1H), 9.5 (s, 1H), 8.5 (d, J = 6.6 Hz, 1H), 8.4 (d, J = 2.0 Hz, 1H), 8.3 (d, J = 9.1 Hz, 1H), 8.2 (d, J = 6.6 Hz, 1H), 8.1 (t, J = 5.6 Hz, 1H), 7.8 (dd, J = 9.0, 2.0 Hz, 1H), 7.6 (dd, J = 8.6, 7.1 Hz, 1H), 7.1 (d, J = 8.6 Hz, 1H), 7.0 (d, J = 6.7 Hz, 2H), 6.6 (t, J = 5.9 Hz, 1H), 5.1 (dd, J = 12.9, 5.4 Hz, 1H), 4.3 (t, J = 8.5 Hz, 1H), 4.2 (dd, J = 8.3, 5.9 Hz, 1H), 4.0 (t, J = 9.0 Hz, 1H), 3.9 (dd, J = 9.3, 5.9 Hz, 1H), 3.8 (d, J = 5.2 Hz, 2H), 3.6 (t, J = 5.4 Hz, 2H), 3.5 (q, J = 5.6 Hz, 4H), 3.4 (q, J = 8.0 Hz, 1H), 3.3 (q, J = 5.7 Hz, 2H), 2.9 (ddd, J = 17.5, 14.0, 5.4 Hz, 1H), 2.6 (td, J = 13.7, 4.1 Hz, 1H), 2.1 – 2.0 (m, 1H). ^{13}C NMR (126 MHz, DMSO-d_6) δ 172.9, 171.4, 170.1, 169.0, 168.5, 167.3, 154.5, 147.2, 146.4, 145.6, 139.9, 136.3, 132.4, 132.1, 131.6, 123.0, 122.9, 122.3, 117.4, 110.7, 109.7, 109.3, 68.8, 68.6, 52.0, 50.5, 48.6, 41.6, 38.7, 32.5, 31.0, 22.2.

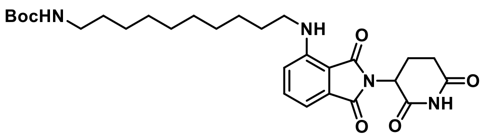

*tert-butyl (10-((2-(2,6-dioxopiperidin-3-yl)-1,3-dioxoisoindolin-4-yl)amino)decyl)carbamate (**6a**).* To a solution of **17** (629 mg, 2.3 mmol) and tert-butyl (10-aminodecyl)carbamate (620 mg, 2.3 mmol) in DMAc (15 mL), was added DIEA (1.2 mL, 6.8 mmol). The mixture was then stirred at 90 °C overnight. After finishing, **6a** was gained via EA extraction, concentration, and silica gel purification (0 - 10% MeOH in DCM, 800 mg, 67% yield). LC-MS (ESI) calculated for $C_{28}H_{40}N_4O_6$ $[M + Na]^+ = 551.29$, found 551.20.

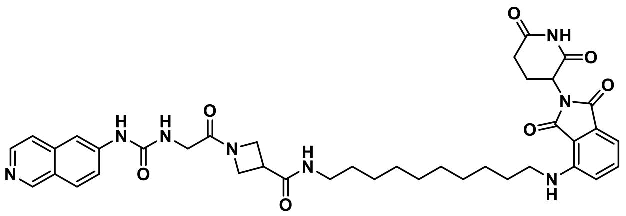

*N-(10-((2-(2,6-dioxopiperidin-3-yl)-1,3-dioxoisoindolin-4-yl)amino)decyl)-1-((isoquinolin-6-ylcarbamoyl)glycyl)azetidine-3-carboxamide (**6**).* Compound **6** was obtained from **6a** using the same method as **4** synthesized, yellow solid (66 mg, 52% yield). HRMS $(M + H)^+$ calcd for $C_{39}H_{47}N_8O_7^+$ 739.3562, found 739.3559. ^{1}H NMR (400 MHz, DMSO-d_6) δ 11.11 (s, 1H), 10.12 (d, J = 5.5 Hz, 1H), 9.55 (s, 1H), 8.45 (d, J = 6.6 Hz, 1H), 8.40 (s, 1H), 8.35 (d, J = 9.0 Hz, 1H), 8.20 (d, J = 6.5 Hz, 1H), 8.00 (dd, J = 14.1, 6.3 Hz, 1H), 7.81 (d, J = 9.0 Hz, 1H), 7.56 (t, J = 7.9 Hz, 1H), 7.10 – 7.02 (m, 2H), 7.00 (d, J = 7.1 Hz, 1H), 6.50 (s, 1H), 5.05 (dd, J = 13.3, 5.3 Hz, 1H), 4.29 (t, J = 8.7 Hz, 1H), 4.17 (t, J = 7.0 Hz, 1H), 3.99 (t, J = 9.1 Hz, 1H), 3.90 (d, J = 7.4 Hz, 1H), 3.77 (d, J = 7.3 Hz, 2H), 3.36 (q, J = 7.3 Hz, 1H), 3.26 (s, 2H), 3.06 (p, J = 6.2 Hz, 2H), 2.95 – 2.82 (m, 1H), 2.63 – 2.52 (m, 2H), 2.04 (dd, J = 15.4, 9.5 Hz, 1H), 1.54 (q, J = 7.3, 6.8 Hz, 2H), 1.43 – 1.13 (m, 16H). ^{13}C NMR (126 MHz, DMSO-d_6) δ 172.9, 171.0, 170.1, 169.0, 168.7, 168.5, 167.3, 154.5, 154.4, 147.4, 146.4, 145.4, 140.1, 136.3, 132.2, 132.1, 131.7, 123.1, 123.0, 117.2, 110.4, 109.0, 52.0, 50.5, 48.6, 42.6, 41.8, 38.7, 38.6, 32.5, 31.0, 29.1, 29.0, 28.8, 28.7, 26.4, 26.3, 22.2.

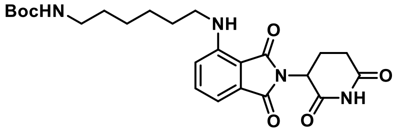

*tert-butyl (6-((2-(2,6-dioxopiperidin-3-yl)-1,3-dioxoisoindolin-4-yl)amino)hexyl)carbamate (7**a**).* To a solution of **17** (1 g, 3.6 mmol) and tert-butyl (6-aminohexyl)carbamate (702 mg, 3.7 mmol) in DMAc (25 mL), was added DIEA (1.8 mL, 10.8 mmol). The mixture was then stirred at 90 °C overnight. After finishing, **7a** was gained via EA extraction, concentration, and silica gel purification (0 - 10% MeOH in DCM, 1.22 g, 71% yield). LC-MS (ESI) calculated for $C_{24}H_{32}N_4O_6$ [M + Na] $^+$ = 495.23, found 495.50.

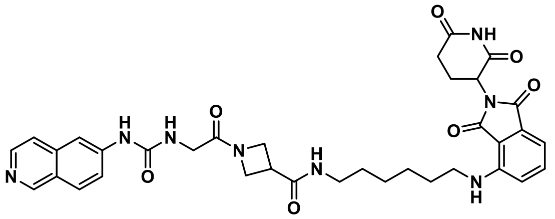

N-(6-((2-(2,6-dioxopiperidin-3-yl)-1,3-dioxoisoindolin-4-yl)amino)hexyl)-1-((isoquinolin-6-ylcarbamoyl)glycyl)azetidine-3-carboxamide (7). Compound **7** was obtained from **7a** using the same method as **4** synthesized, yellow solid (66 mg, 52% yield). HRMS $(M + H)^+$ calcd for $C_{35}H_{39}N_8O_7^+$ 683.2936, found 683.2935. ^{1}H NMR (400 MHz, DMSO-d_6) δ 11.1 (s, 1H), 10.1 (s, 1H), 9.5 (s, 1H), 8.4 (d, J = 6.5 Hz, 1H), 8.4 (s, 1H), 8.3 (d, J = 9.1 Hz, 1H), 8.2 (d, J = 6.5 Hz, 1H), 8.1 (t, J = 5.6 Hz, 1H), 7.8 – 7.8 (m, 1H), 7.6 (t, J = 7.8 Hz, 1H), 7.1 (d, J = 8.6 Hz, 2H), 7.0 (d, J = 7.0 Hz, 1H), 6.5 (s, 1H), 5.0 (dd, J = 12.8, 5.3 Hz, 1H), 4.3 (t, J = 8.5 Hz, 1H), 4.2 – 4.1 (m, 1H), 4.0 (t, J = 9.0 Hz, 1H), 3.9 (dd, J = 9.3, 5.9 Hz, 1H), 3.8 (d, J = 5.1 Hz, 2H), 3.4 (td, J = 8.7, 4.4 Hz, 1H), 3.3 (d, J = 7.8 Hz, 2H), 3.1 (q, J = 6.5 Hz, 2H), 2.9 (ddd, J = 18.2, 14.0, 5.3 Hz, 1H), 2.6 – 2.5 (m, 2H), 2.0 (dq, J = 12.9, 5.9, 5.2 Hz, 1H), 1.6 – 1.5 (m, 2H), 1.4 – 1.2 (m, 6H). ^{13}C NMR (126 MHz, DMSO-d_6) δ 172.9, 171.0, 170.2, 169.0, 168.5, 167.3, 154.5, 147.3, 146.4, 145.5, 140.0, 136.3, 132.3, 132.2, 131.6, 123.0, 122.9, 122.3, 117.2, 110.4, 109.7, 109.0, 52.1, 50.5, 48.6, 41.8, 38.7, 32.5, 31.0, 28.9, 28.6, 26.1, 26.0, 22.2.

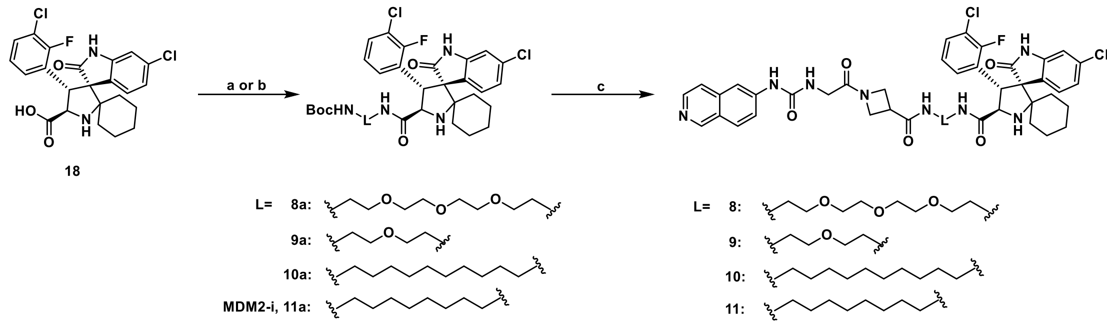

Scheme S5. **Reaction conditions:** (a) amino-acid linkers, NMM, HOBt, EDCI, rt., 3 h; (b) amino-acid linkers, DIEA, HATU, DMF, rt., 3 h. (c) (i) TFA, DCM, rt., 1 h; (ii) **15**, DIEA, HATU, DMF, rt., 3 h. Compound **18** was synthesized according to published procedures.[4]

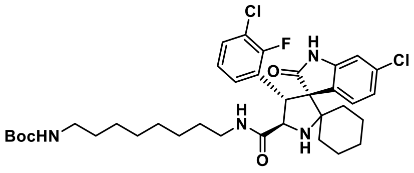

tert-butyl(8-((3'R,4'S,5'R)-6''-chloro-4'-(3-chloro-2-fluorophenyl)-2''-oxodispiro[cyclohexane-1,2'-pyrrolidine-3',3''-indoline]-5'-carboxamido)octyl)carbamate ***(11a, MDM2-i)****.* To a solution of **18** (50 mg, 0.11 mmol) and tert-butyl (8-aminooctyl)carbamate (26 mg, 0.11 mmol) in DMF (3 mL), was added EDCI (31 mg, 0.16 mmol), NMM (30 μL, 0.28 mmol) followed by HOBt (22 mg, 0.16 mmol). The mixture was stirred for 3 h remaining room temperature. After finishing, **MDM2-i** was gained via EA extraction, concentration, and silica gel purification (0 - 4% MeOH in DCM, 44 mg, 59% yield). LC-MS (ESI) calculated for $C_{36}H_{47}Cl_2FN_4O_7$ [M + H] $^+$ = 689.30, found 689.17.

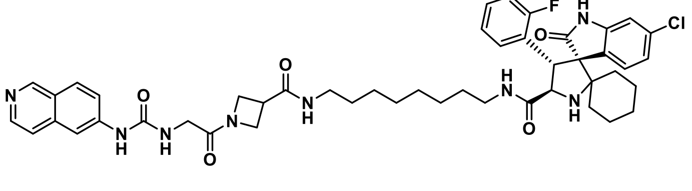

(3'R,4'S,5'R)-6''-chloro-4'-(3-chloro-2-fluorophenyl)-N-(8-(1-((isoquinolin-6-ylcarbamoyl) glycyl)azetidine-3-carboxamido)octyl)-2''-oxodispiro[cyclohexane-1,2'-pyrrolidine-3',3''-indoline]-5'-carboxamide ***(11)****.* **MDM2-*i*** (40 mg, 0.06 mmol) was dissolved in DCM : TFA (5 mL : 1 mL) and the mixture was stirred at room temperature for 0.5 h. The reaction was then concentrated under reduced pressure to give deprotection intermediate which was used for the next step without

purification. To a solution of deprotection intermediate and **15** (19 mg, 0.06 mmol) in DMF (3 mL), DIEA (0.05 mL, 0.3 mmol) was added followed by HATU (28 mg, 0.08 mmol). The mixture was stirred at room temperature for 3 h. After finishing, the mixture was purified by C18 reverse phase preparative high-performance liquid chromatography to give the final compound **11** (white solid, 20 mg, 38% yield). HRMS $(M + H)^+$ calcd for $C_{47}H_{54}Cl_2FN_8O_5^+$ 899.3573, found 899.3576. ^1H NMR (400 MHz, DMSO-d_6) δ 11.07 (s, 1H), 10.14 (s, 1H), 9.55 (s, 1H), 8.56 (t, J = 5.8 Hz, 1H), 8.46 (d, J = 6.6 Hz, 1H), 8.40 (d, J = 2.0 Hz, 1H), 8.35 (d, J = 9.0 Hz, 1H), 8.19 (d, J = 6.6 Hz, 1H), 8.03 (t, J = 5.6 Hz, 1H), 7.81 (dd, J = 9.1, 2.1 Hz, 1H), 7.61 (t, J = 7.2 Hz, 1H), 7.53 (dd, J = 8.1, 2.6 Hz, 1H), 7.44 (t, J = 7.6 Hz, 1H), 7.21 (t, J = 8.0 Hz, 1H), 7.11 – 7.04 (m, 2H), 6.76 (d, J = 2.0 Hz, 1H), 4.97 (d, J = 10.8 Hz, 1H), 4.65 (d, J = 10.8 Hz, 1H), 4.29 (t, J = 8.5 Hz, 1H), 4.17 (dd, J = 8.2, 5.8 Hz, 1H), 3.99 (t, J = 9.0 Hz, 1H), 3.88 (dd, J = 9.3, 5.9 Hz, 1H), 3.79 (d, J = 5.2 Hz, 2H), 3.36 (tt, J = 8.9, 5.8 Hz, 1H), 3.21 (dt, J = 13.2, 6.6 Hz, 1H), 3.05 (q, J = 6.6 Hz, 2H), 2.89 (dq, J = 12.0, 5.8 Hz, 1H), 2.60 (d, J = 12.9 Hz, 1H), 1.94 (q, J = 14.8, 13.7 Hz, 2H), 1.58 (ddt, J = 50.6, 27.0, 12.2 Hz, 5H), 1.36 (p, J = 7.1 Hz, 2H), 1.27 – 0.96 (m, 11H), 0.85 (p, J = 7.3 Hz, 2H). HPLC retention time 6.31 min, > 98% pure.

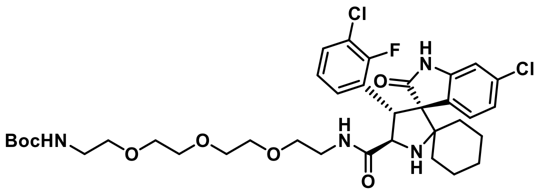

*tert-butyl (1-((3'R,4'S,5'R)-6''-chloro-4'-(3-chloro-2-fluorophenyl)-2''-oxodispiro[cyclohexane-1,2'-pyrrolidine-3',3''-indolin]-5'-yl)-1-oxo-5,8,11-trioxa-2-azatridecan-13-yl)carbamate (**8a**).* To a solution of **18** (90 mg, 0.2 mmol) and tert-butyl (2-(2-(2-(2-aminoethoxy)ethoxy) ethoxy)ethyl)carbamate (57 mg, 0.2 mmol) in DMF (3 mL), was added EDCI (56 mg, 0.3 mmol), NMM (43 µL, 0.4 mmol) followed by HOBt (40 mg, 0.3 mmol). The mixture was stirred for 3 h remaining room temperature. After finishing, **8a** was gained via EA extraction, concentration, and silica gel purification (0 - 5% MeOH in DCM, 89 mg, 62% yield). LC-MS (ESI) calculated for $C_{36}H_{47}Cl_2FN_4O_7$ $[M + H]^+$ = 737.28, found 737.11.

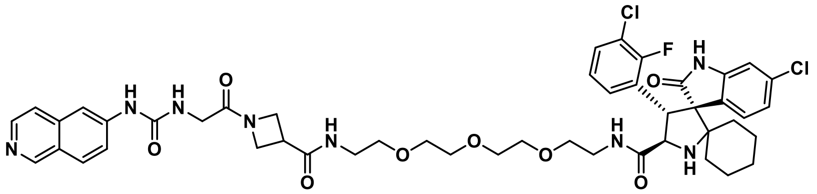

(3'R,4'S,5'R)-6''-chloro-4'-(3-chloro-2-fluorophenyl)-N-(1-(1-((isoquinolin-6-ylcarbamoyl) glycyl)azetidin-3-yl)-1-oxo-5,8,11-trioxa-2-azatridecan-13-yl)-2''-oxodispiro[cyclohexane-1,2'-pyrrolidine-3',3''-indoline]-5'-carboxamide (8). Compound **8** was obtained from **8a** by using the same method as **11** synthesized, white solid (38 mg, 36% yield). HRMS $(M + H)^+$ calcd for $C_{47}H_{54}Cl_2FN_8O_8^+$ 947.3420, found 947.3418. ^1H NMR (400 MHz, DMSO-d_6) δ 11.0 (s, 1H), 10.1 (s, 1H), 9.6 (s, 1H), 8.7 (s, 1H), 8.5 (d, J = 6.6 Hz, 1H), 8.4 (s, 1H), 8.4 (d, J = 9.1 Hz, 1H), 8.2 (d, J = 6.5 Hz, 1H), 8.1 (t, J = 5.6 Hz, 1H), 7.8 (dd, J = 9.1, 2.0 Hz, 1H), 7.6 (t, J = 7.3 Hz, 1H), 7.5 (dd, J = 8.2, 2.5 Hz, 1H), 7.4 (t, J = 7.6 Hz, 1H), 7.2 (t, J = 8.2 Hz, 1H), 7.1 – 7.0 (m, 2H), 6.8 (d, J = 2.0 Hz, 1H), 5.0 (d, J = 10.8 Hz, 1H), 4.7 (d, J = 10.7 Hz, 1H), 4.3 (t, J = 8.5 Hz, 1H), 4.2 (dd, J = 8.2, 5.9 Hz, 1H), 4.0 (t, J = 8.9 Hz, 1H), 3.9 (dd, J = 9.2, 5.9 Hz, 1H), 3.8 (d, J = 5.0 Hz, 2H), 3.5 – 3.2 (m, 17H), 3.1 (q, J = 8.6, 8.0 Hz, 1H), 2.6 (d, J = 12.9 Hz, 1H), 1.9 (t, J = 12.6 Hz, 2H), 1.7 – 1.4 (m, 5H), 1.0 (td, J = 13.4, 4.5 Hz, 2H). ^{13}C NMR (126 MHz, DMSO-d_6) δ 176.3, 171.3, 168.4, 155.6 (d, $J_{C\text{-}F}$ = 248.8 Hz), 154.4, 147.5, 145.3, 144.0, 140.1, 134.0, 131.8, 130.5, 128.6, 125.3, 123.1 (d, $J_{C\text{-}F}$ = 13.2 Hz), 122.2, 121.5, 119.4 (d, $J_{C\text{-}F}$= 18.8 Hz), 110.0, 109.7, 70.7, 69.7, 69.6, 69.5, 69.5, 68.9, 68.7, 66.5, 60.0, 51.9, 50.5, 44.9, 38.7, 34.8, 32.4, 30.9, 30.1, 24.0, 21.5, 20.2. HPLC retention time 5.90 min, > 96% pure.

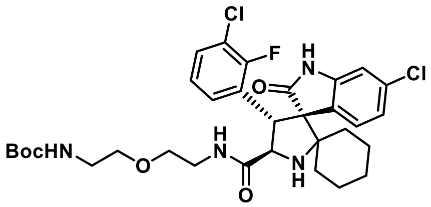

tert-butyl (2-(2-((3'R,4'S,5'R)-6''-chloro-4'-(3-chloro-2-fluorophenyl)-2''-oxodispiro[cyclohexane-1,2'-pyrrolidine-3',3''-indoline]-5'-carboxamido)ethoxy)ethyl)carbamate (9a). To a solution of **18** (90 mg, 0.2 mmol) and tert-butyl (2-(2-aminoethoxy)ethyl)carbamate (40 mg, 0.2 mmol) in DMF (3 mL), was added EDCI (56 mg, 0.3 mmol), NMM (43 μL, 0.4 mmol) followed by HOBt (40 mg, 0.3 mmol). The mixture was stirred for 3 h remaining room temperature. After finishing, **9a** was gained via EA extraction, concentration, and silica gel purification (0 - 5% MeOH in DCM, 84 mg,

67% yield). LC-MS (ESI) calculated for $C_{32}H_{39}Cl_2FN_4O_5$ $[M + H]^+$ = 649.23, found 649.15.

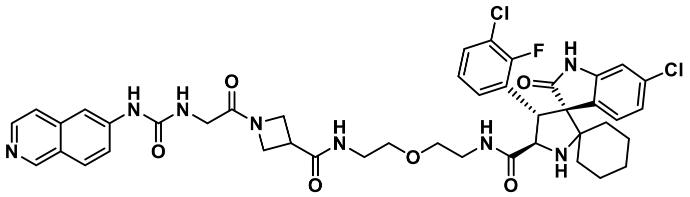

*(3'R,4'S,5'R)-6''-chloro-4'-(3-chloro-2-fluorophenyl)-N-(2-(2-(1-((isoquinolin-6-ylcarbamoyl) glycyl)azetidine-3-carboxamido)ethoxy)ethyl)-2''-oxodispiro[cyclohexane-1,2'-pyrrolidine-3',3''-indoline]-5'-carboxamide (**9**)*. Compound **9** was obtained from **9a** using the same method as **11** synthesized, white solid (35 mg, 33% yield). HRMS $(M + H)^+$ calcd for $C_{43}H_{46}Cl_2FN_8O_6^+$ 859.2896, found 859.2894. ^{1}H NMR (400 MHz, DMSO-d_6) δ 10.9 (s, 1H), 10.1 (s, 1H), 9.6 (s, 1H), 8.6 (s, 1H), 8.5 (s, 1H), 8.4 – 8.3 (m, 2H), 8.2 (d, *J* = 6.3 Hz, 1H), 8.1 (t, *J* = 5.6 Hz, 1H), 7.8 (dd, *J* = 9.1, 2.1 Hz, 1H), 7.6 (t, *J* = 7.2 Hz, 1H), 7.5 (dd, *J* = 8.1, 2.6 Hz, 1H), 7.4 (t, *J* = 7.5 Hz, 1H), 7.2 (t, *J* = 8.1 Hz, 1H), 7.1 (dd, *J* = 8.1, 2.0 Hz, 1H), 7.0 (t, *J* = 5.3 Hz, 1H), 6.7 (d, *J* = 2.1 Hz, 1H), 4.9 (d, *J* = 10.4 Hz, 1H), 4.6 (d, *J* = 10.4 Hz, 1H), 4.3 – 4.2 (m, 1H), 4.2 (dd, *J* = 8.3, 5.8 Hz, 1H), 4.0 (td, *J* = 9.1, 3.2 Hz, 1H), 3.9 (ddd, *J* = 8.8, 5.9, 2.3 Hz, 1H), 3.8 (d, *J* = 5.1 Hz, 2H), 3.4 – 3.1 (m, 9H), 2.5 – 2.4 (m, 1H), 1.9 (t, *J* = 13.0 Hz, 2H), 1.7 – 1.4 (m, 5H), 1.0 (t, *J* = 12.8 Hz, 2H). ^{13}C NMR (126 MHz, DMSO-d_6) δ 176.4, 171.0, 168.5, 155.6 (d, $J_{C\text{-}F}$ = 248.9 Hz), 154.5, 147.3, 145.6, 144.0, 140.0, 134.0, 132.4, 131.7, 130.6, 128.6, 125.3 (d, $J_{C\text{-}F}$ = 4.5 Hz), 123.1, 122.9, 122.3 (d, $J_{C\text{-}F}$ = 15.2 Hz), 121.5, 119.5 (d, $J_{C\text{-}F}$ = 18.9 Hz), 110.0, 109.7, 70.7, 66.4, 60.3, 52.0, 50.5, 45.1, 38.7, 38.7, 32.5, 31.1, 30.2, 29.0, 28.6, 28.6, 28.5, 26.4, 25.8, 24.1, 21.6, 20.3. HPLC retention time 5.88 min, > 97% pure.

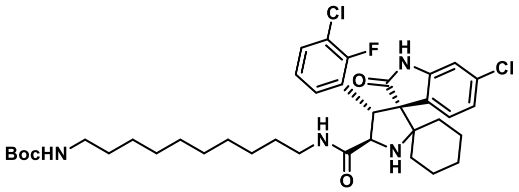

*tert-butyl (10-((3'R,4'S,5'R)-6''-chloro-4'-(3-chloro-2-fluorophenyl)-2''-oxodispiro[cyclohexane-1,2'-pyrrolidine-3',3''-indoline]-5'-carboxamido)decyl)carbamate (**10a**)*. To a solution of **18** (50 mg, 0.1 mmol) and tert-butyl (10-aminodecyl)carbamate (30 mg, 0.1 mmol) in DMF (3 mL), was added DIEA (0.1 mL, 0.5 mmol), followed by HATU (53 mg, 0.13 mmol). The mixture was stirred for 3

h remaining room temperature. After finishing, **10a** was gained via EA extraction, concentration, and silica gel purification (0 - 5% MeOH in DCM, 50 mg, 67% yield). LC-MS (ESI) calculated for $C_{38}H_{51}Cl_2FN_4O_4$ [M + H] $^+$ = 717.33, found 717.22.

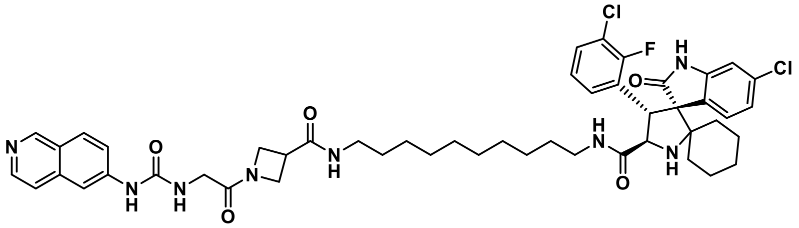

*(3'R,4'S,5'R)-6''-chloro-4'-(3-chloro-2-fluorophenyl)-N-(10-(1-((isoquinolin-6-ylcarbamoyl) glycyl)azetidine-3-carboxamido)decyl)-2''-oxodispiro[cyclohexane-1,2'-pyrrolidine-3',3''-indoline]-5'-carboxamide (**10**)*. Compound **10** was obtained from **10a** using the same method as **11** synthesized, white solid (23 mg, 20% yield). HRMS $(M + H)^+$ calcd for $C_{49}H_{58}Cl_2FN_8O_5^+$ 927.3886, found 927.3888. ^{1}H NMR (400 MHz, DMSO-d_6) δ 11.0 (d, J = 9.8 Hz, 1H), 10.1 (s, 1H), 9.6 (s, 1H), 8.6 (s, 1H), 8.5 (s, 1H), 8.4 (s, 1H), 8.4 (d, J = 9.1 Hz, 1H), 8.2 (d, J = 6.3 Hz, 1H), 8.0 (d, J = 5.8 Hz, 1H), 7.8 (dd, J = 9.2, 2.1 Hz, 1H), 7.6 (t, J = 7.3 Hz, 1H), 7.5 (dd, J = 8.2, 2.5 Hz, 1H), 7.5 – 7.4 (m, 1H), 7.2 (t, J = 8.0 Hz, 1H), 7.1 (dd, J = 8.2, 2.0 Hz, 1H), 6.9 (s, 1H), 6.7 (d, J = 2.1 Hz, 1H), 5.0 (d, J = 11.4 Hz, 1H), 4.6 (d, J = 10.9 Hz, 1H), 4.3 (t, J = 8.5 Hz, 1H), 4.2 (dd, J = 8.2, 5.8 Hz, 1H), 4.0 (t, J = 9.0 Hz, 1H), 3.9 (dd, J = 9.2, 5.9 Hz, 1H), 3.8 (d, J = 5.1 Hz, 2H), 3.3 (ddd, J = 8.9, 5.9, 2.9 Hz, 1H), 3.2 (dt, J = 13.4, 6.7 Hz, 1H), 3.1 (q, J = 6.5 Hz, 2H), 2.9 (dd, J = 13.1, 6.1 Hz, 1H), 2.6 (d, J = 18.1 Hz, 1H), 2.0 (q, J = 13.6, 10.9 Hz, 2H), 1.7 – 1.3 (m, 7H), 1.3 – 1.0 (m, 15H), 0.9 – 0.8 (m, 2H). ^{13}C NMR (126 MHz, DMSO-d_6) δ 176.3, 171.0, 168.4, 155.6 (d, $J_{C\text{-}F}$ = 248.9 Hz), 154.4, 147.37, 145.5, 144.0, 140.0, 134.1, 131.8, 130.7, 128.6 (d, $J_{C\text{-}F}$ = 14.4 Hz), 125.4, 123.1, 121.5, 119.6 (d, $J_{C\text{-}F}$ = 18.7 Hz), 110.0, 109.7, 70.8, 66.28, 60.17, 52.0, 50.5, 45.1, 38.7, 32.5, 31.0, 30.1, 29.0, 29.0, 28.9, 28.7, 28.6, 28.5, 26.4, 25.8, 24.0, 21.5, 20.2. HPLC retention time 6.88 min, > 99% pure.

NMR spectra of synthesized compounds

Intermediate 15 ^{1}H NMR

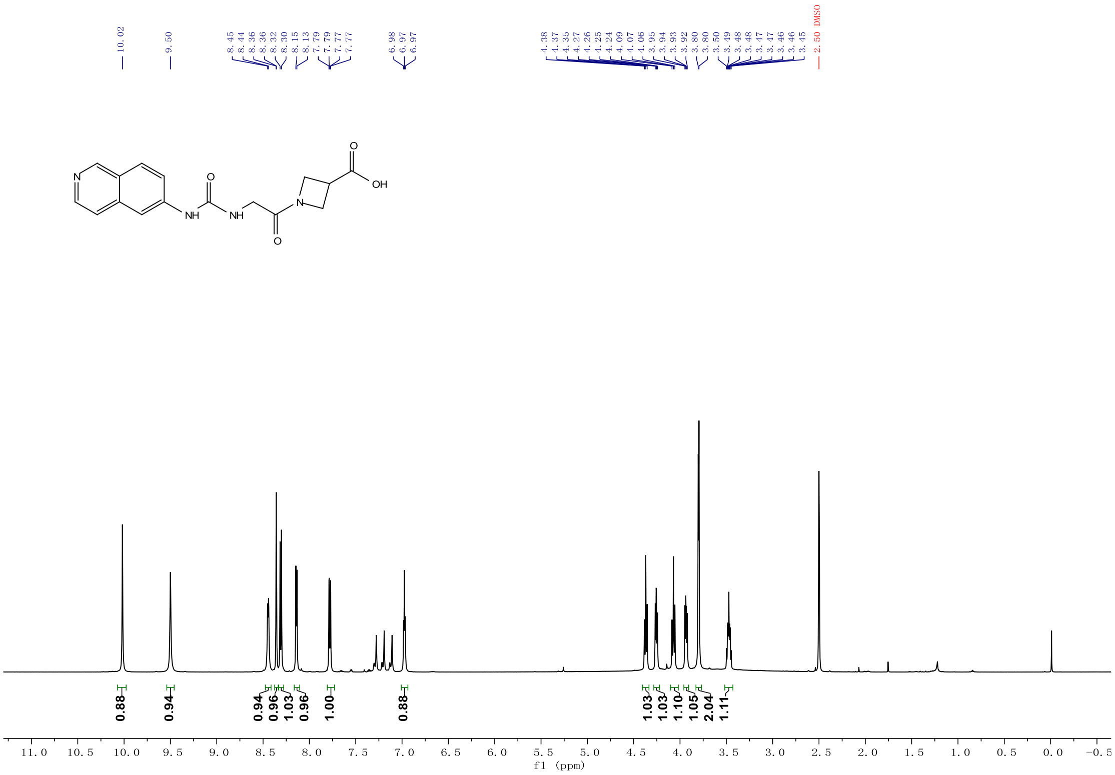

Intermediate 15 ^{13}C NMR

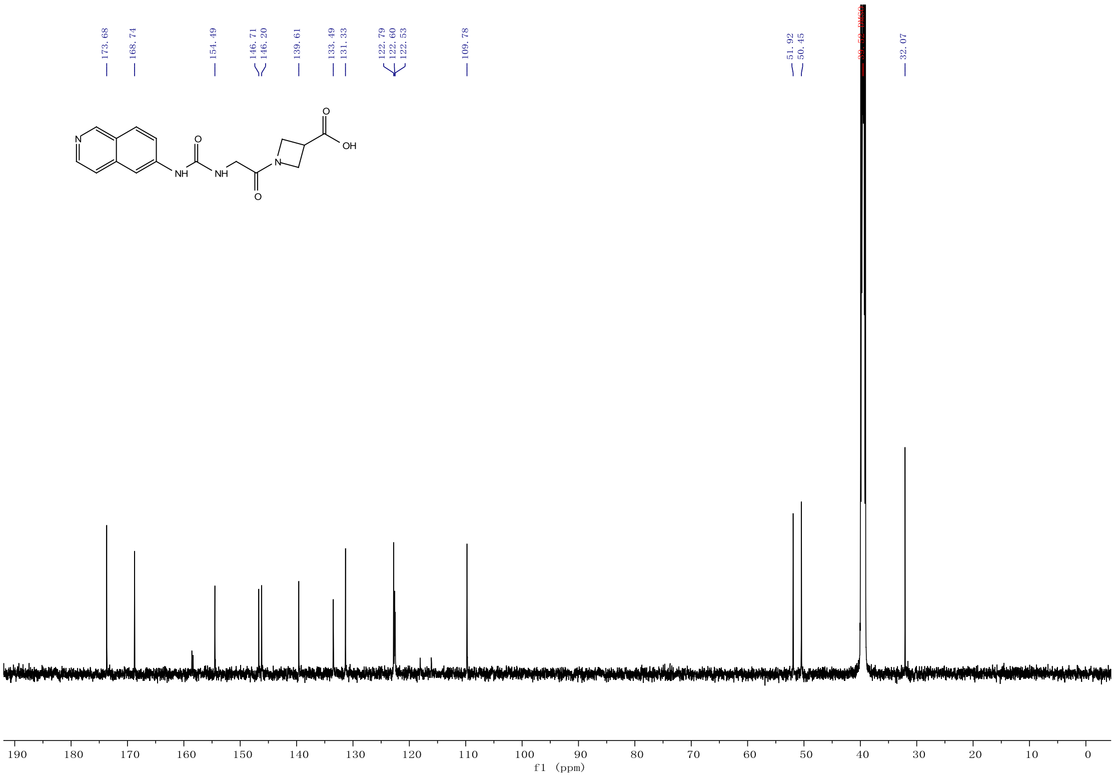

Compound 1 ^{1}H NMR

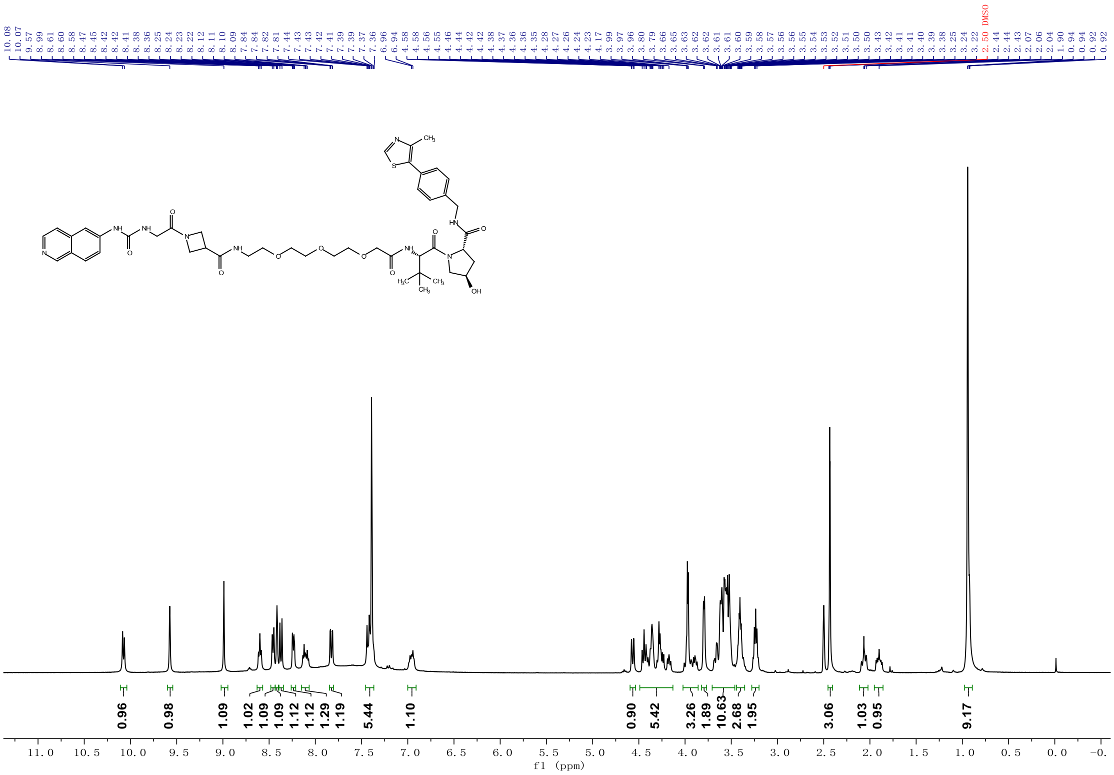

Compound 1 ^{13}C NMR

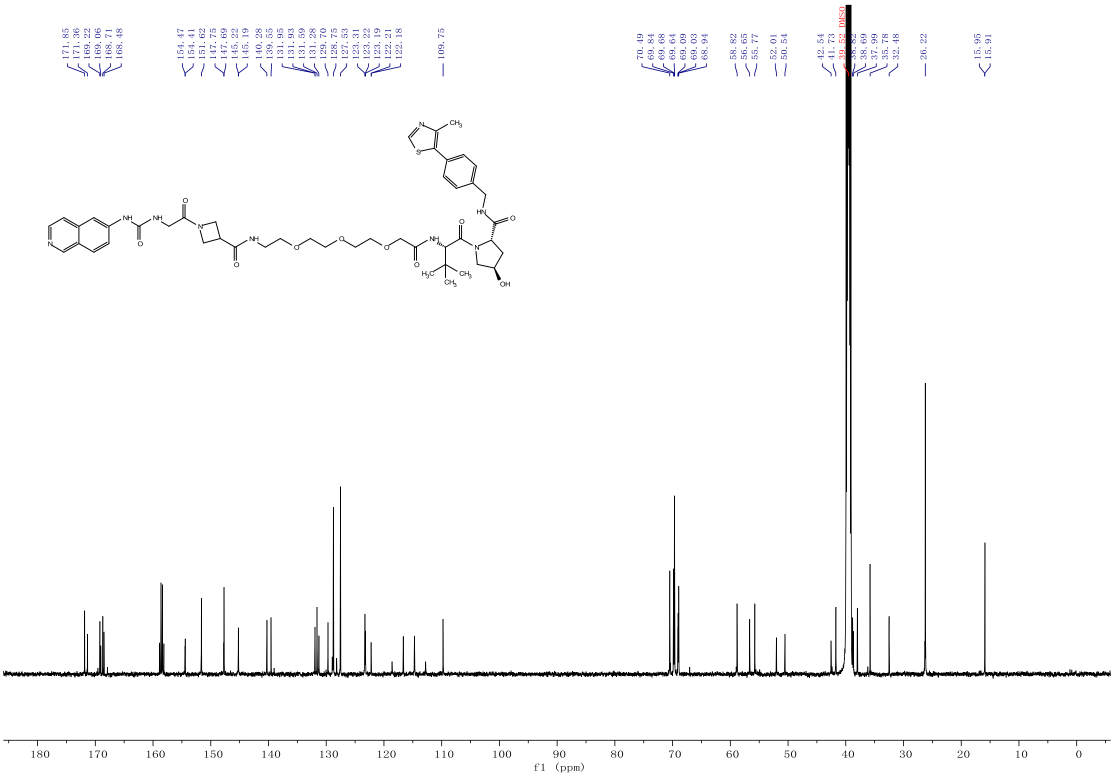

Compound 2 ^{1}H NMR

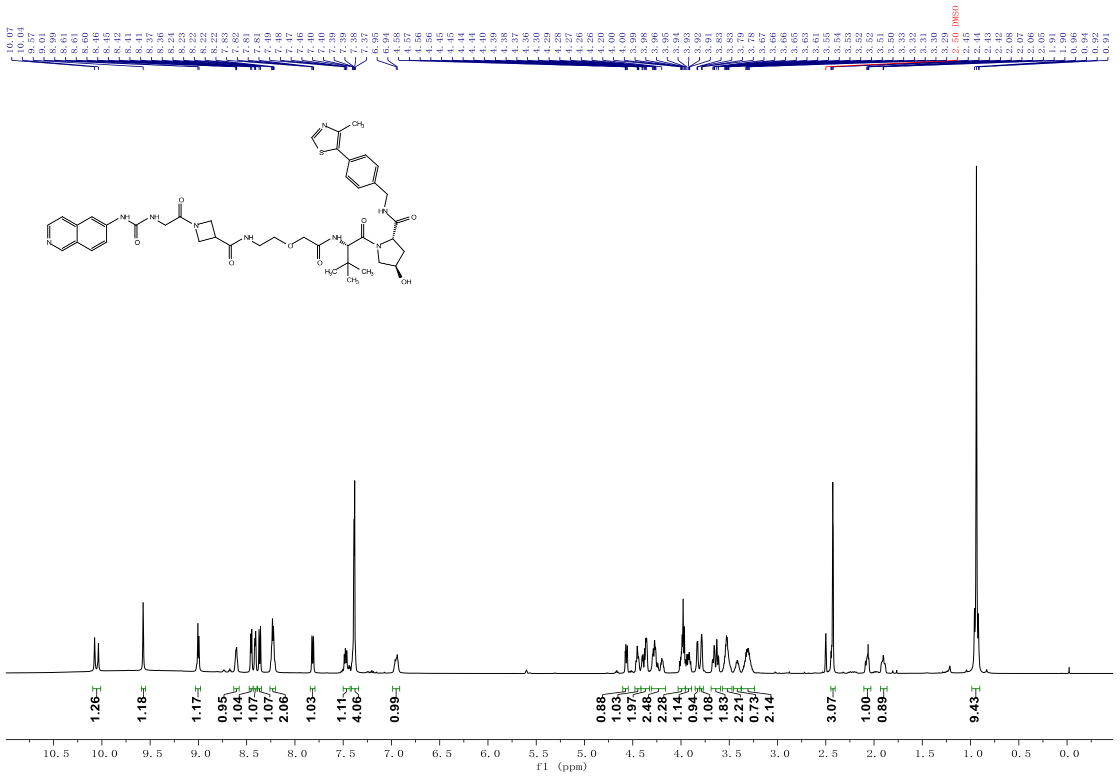

Compound 2 ^{13}C NMR

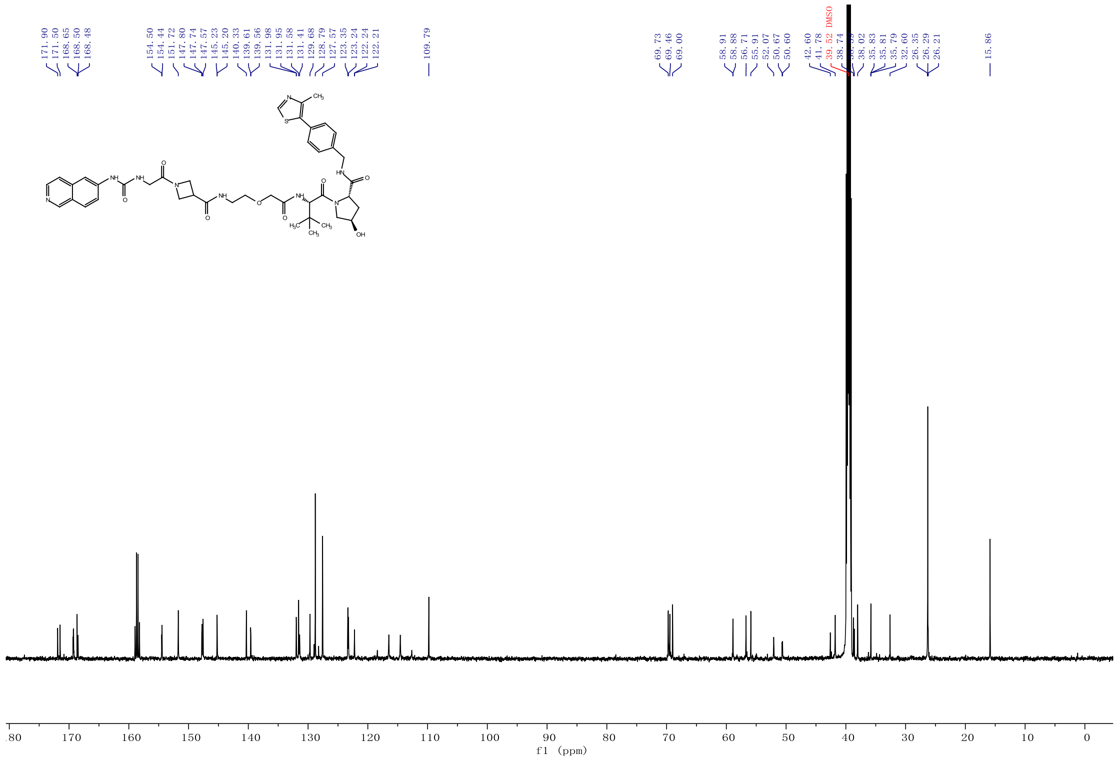

Compound 3 ^{1}H NMR

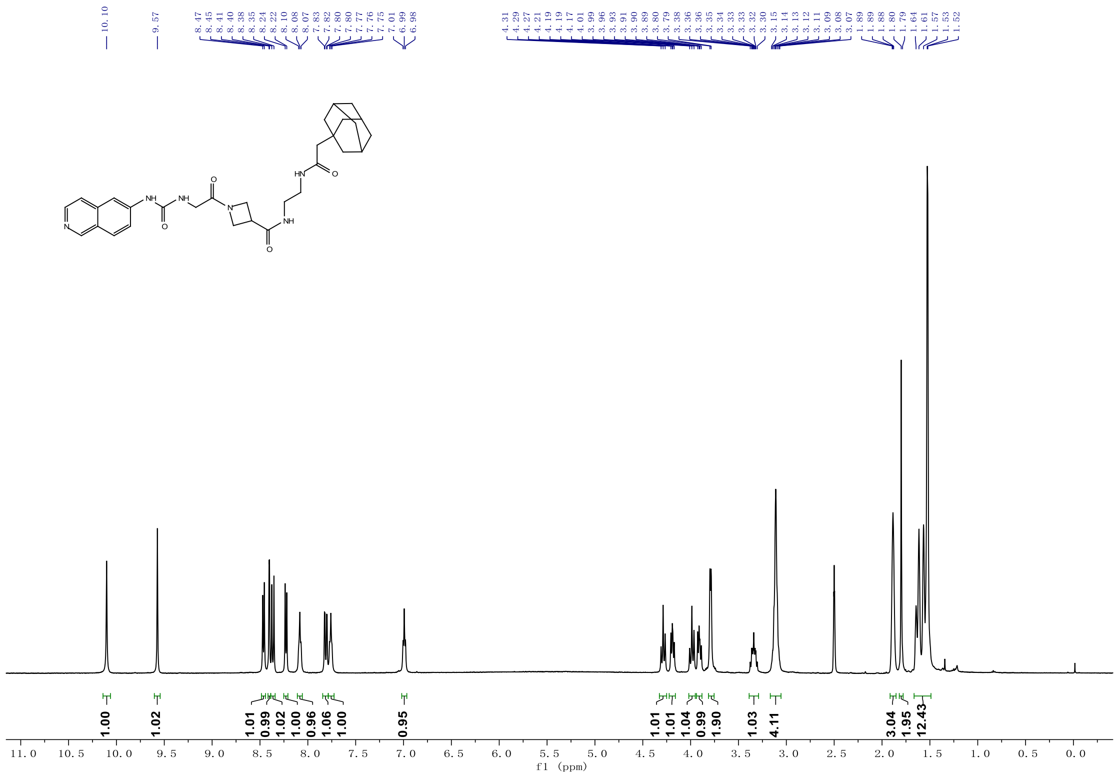

Compound 3 ^{13}C NMR

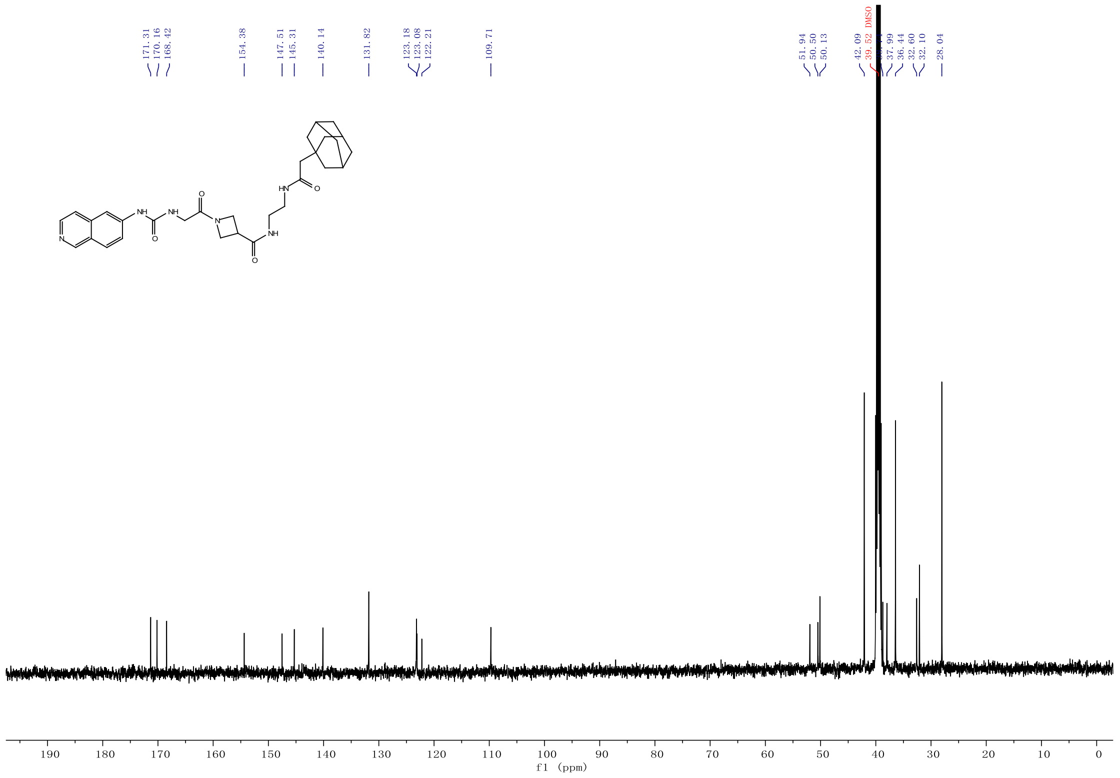

Compound 4 ^{1}H NMR

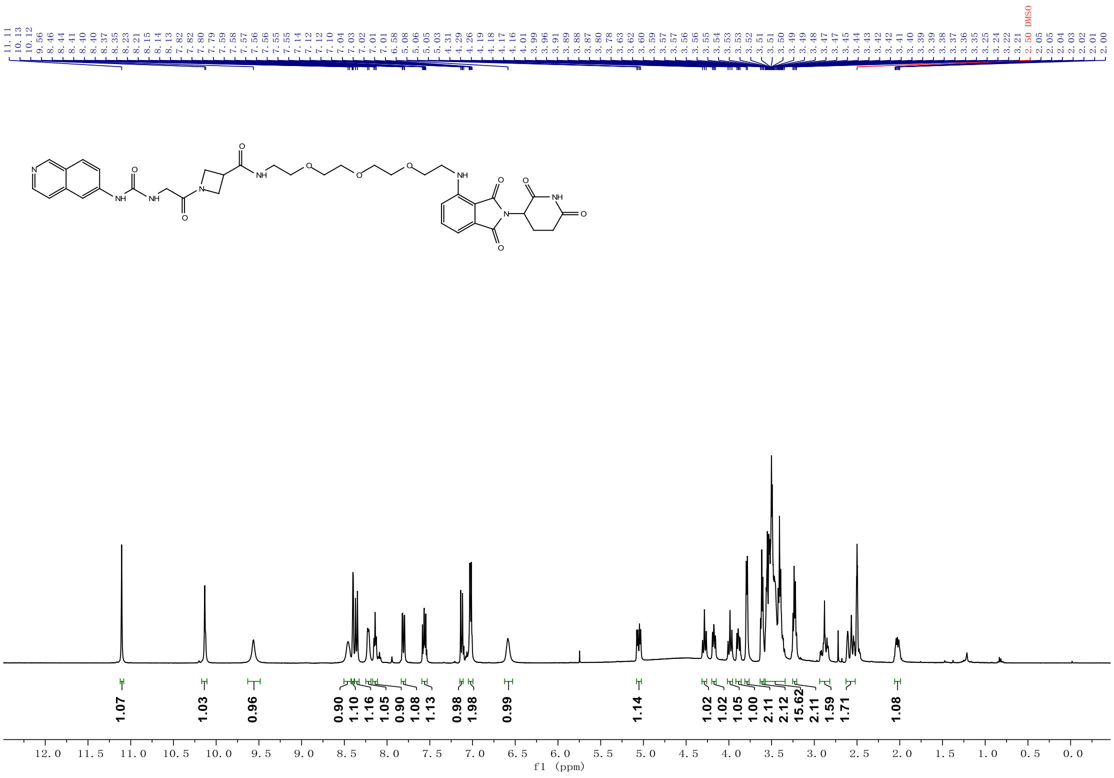

Compound 4 ^{13}C NMR

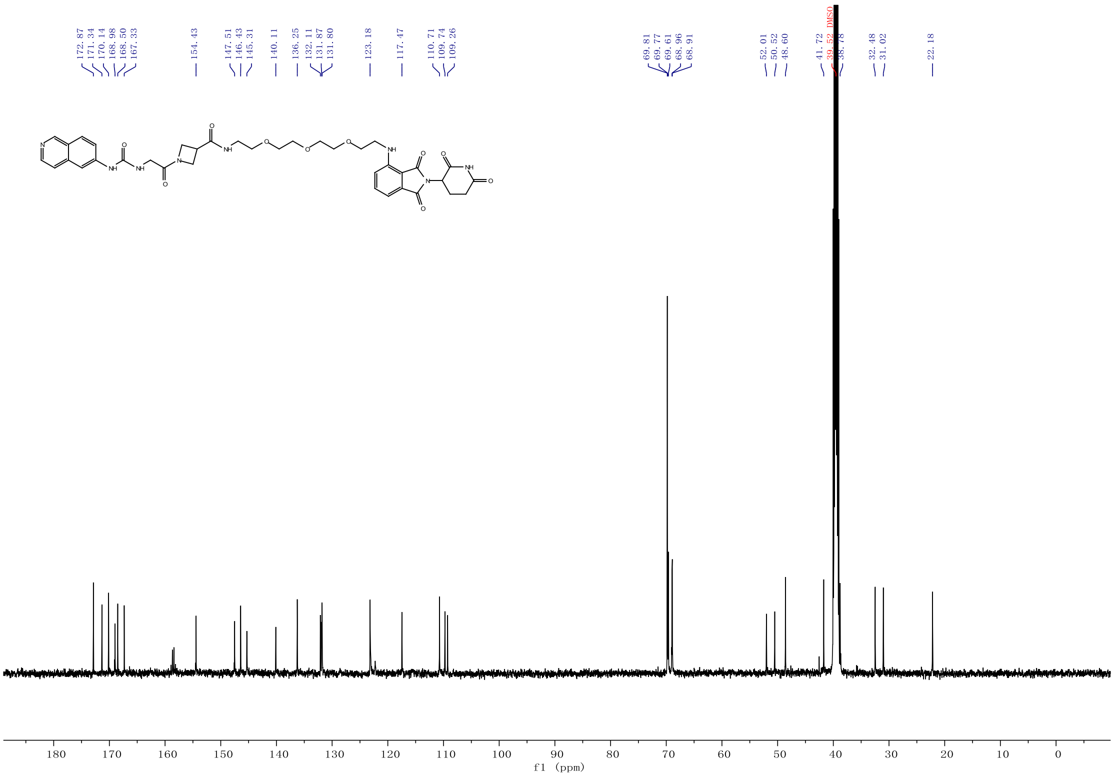

Compound 5 ^{1}H NMR

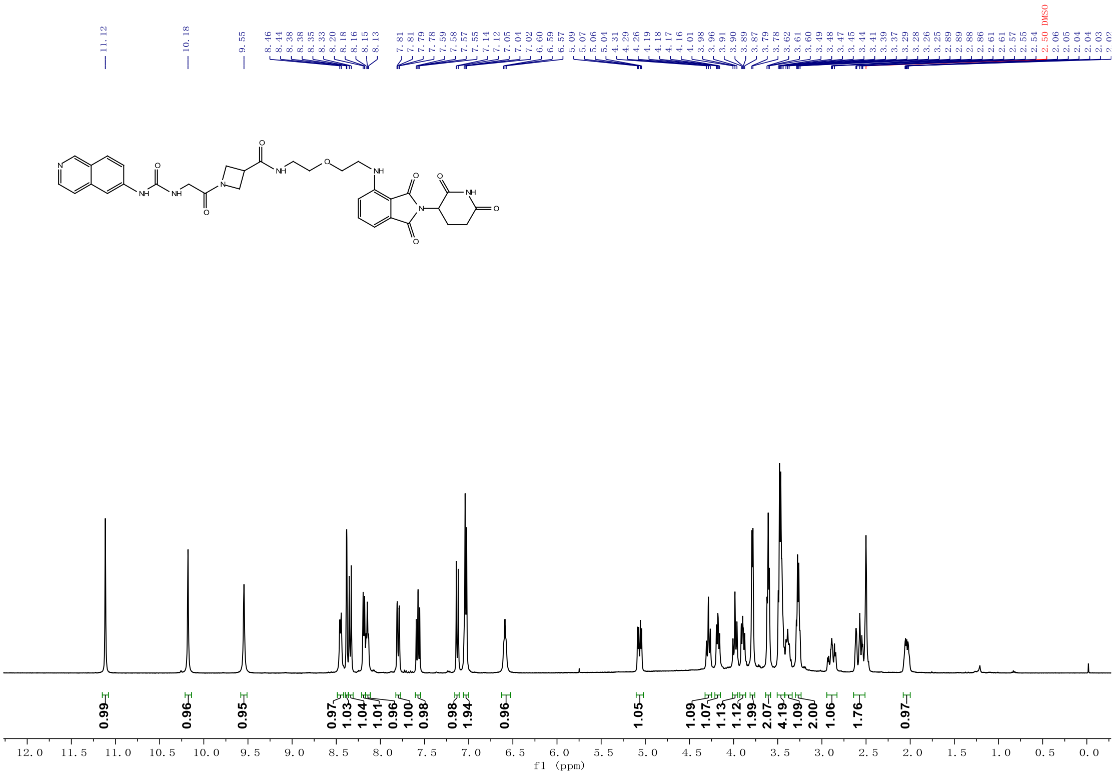

Compound 5 ^{13}C NMR

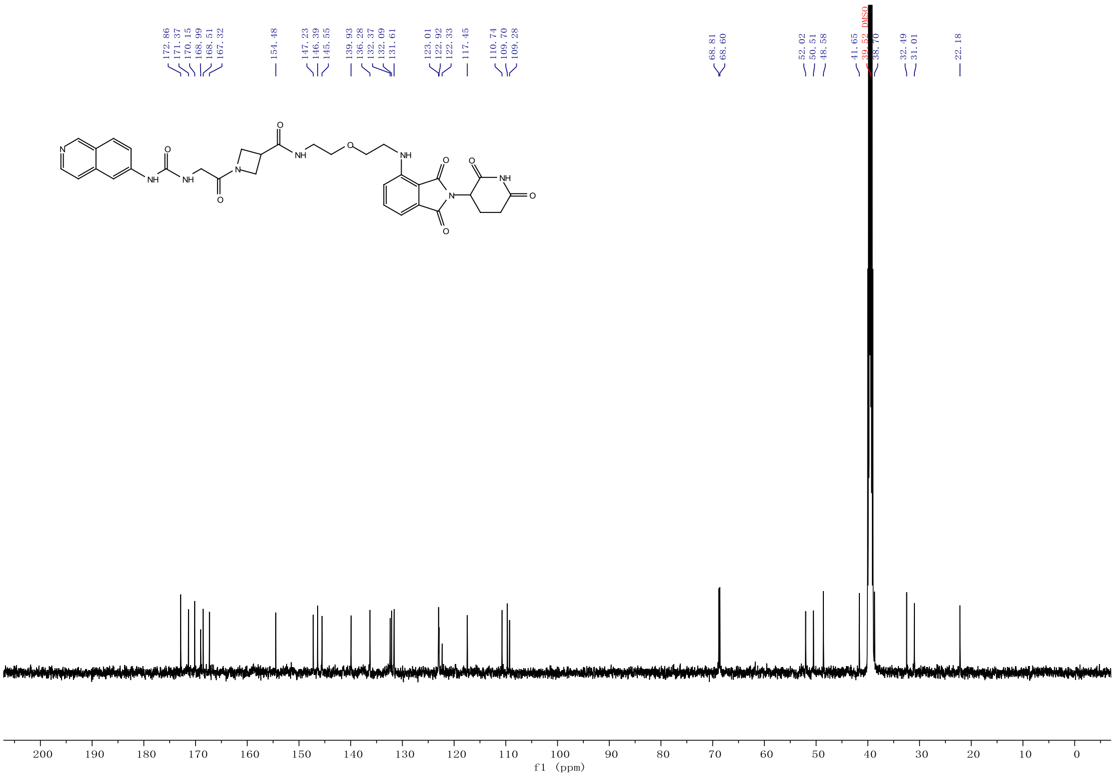

Compound 6 ^{1}H NMR

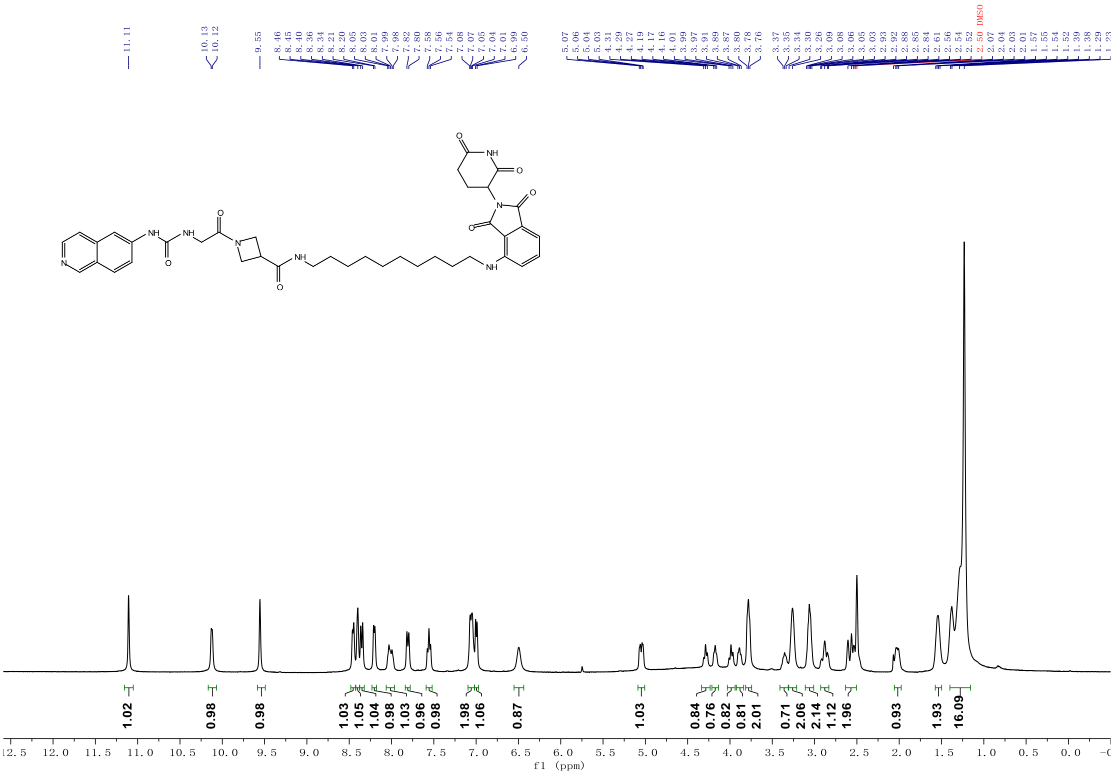

Compound 6 ^{13}C NMR

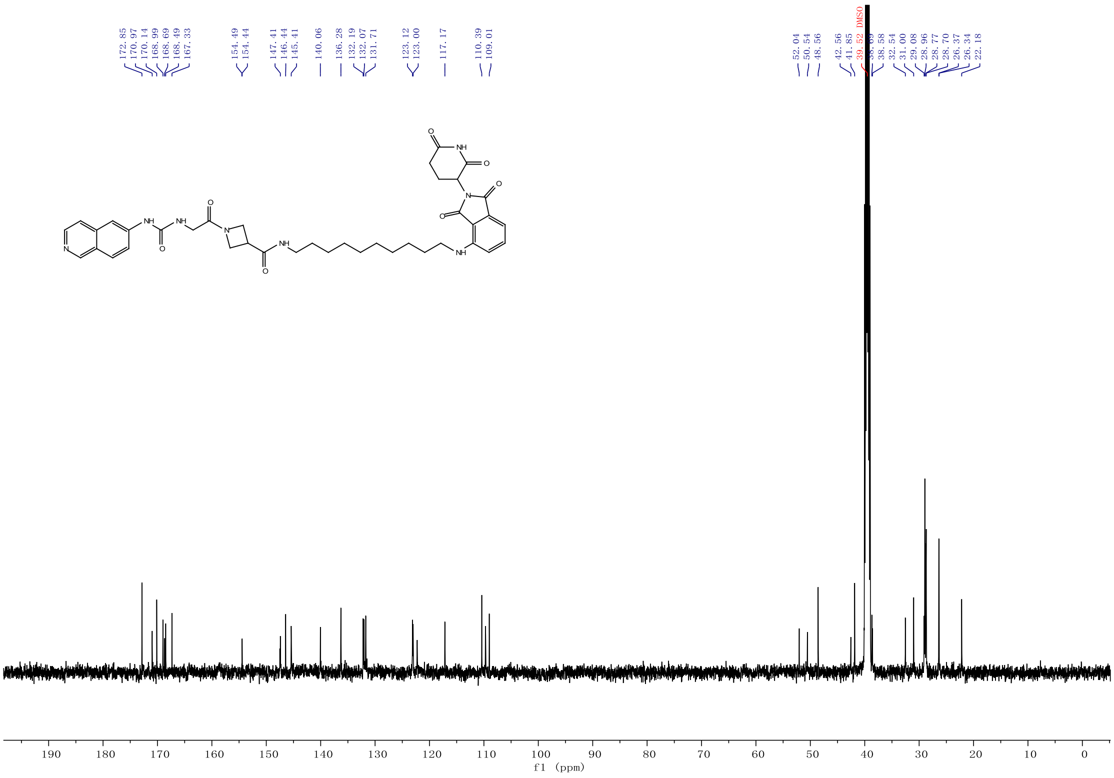

Compound 7 ^{1}H NMR

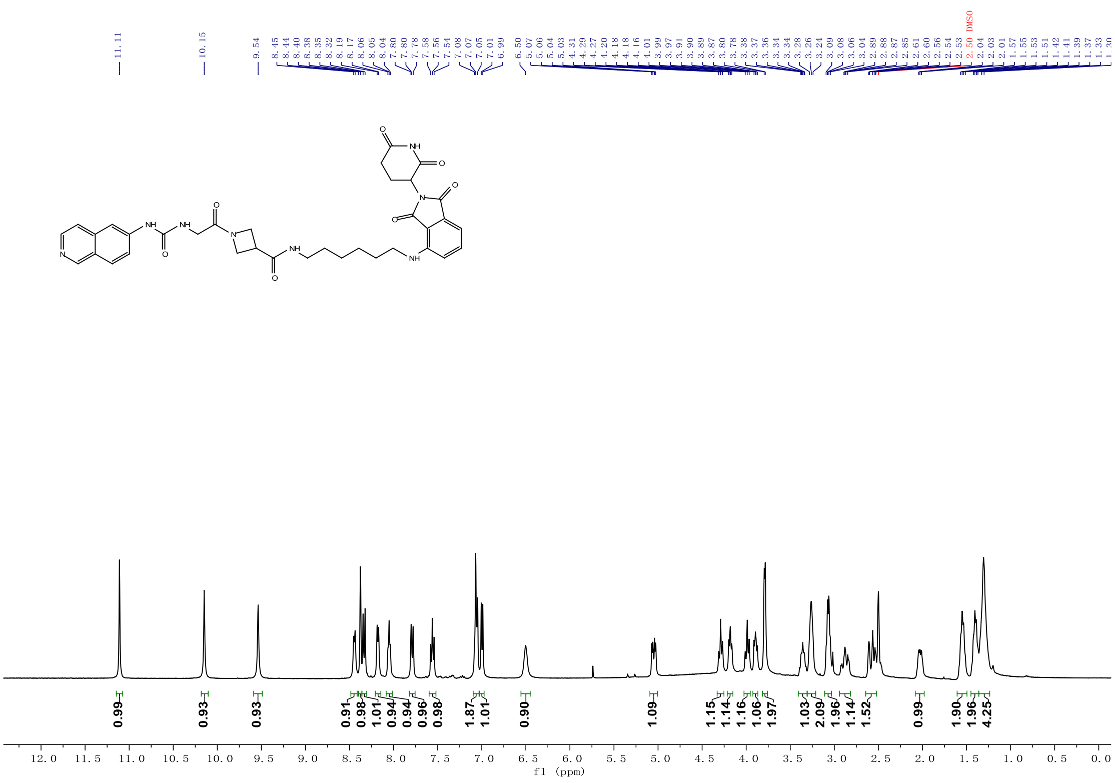

Compound 7 ^{13}C NMR

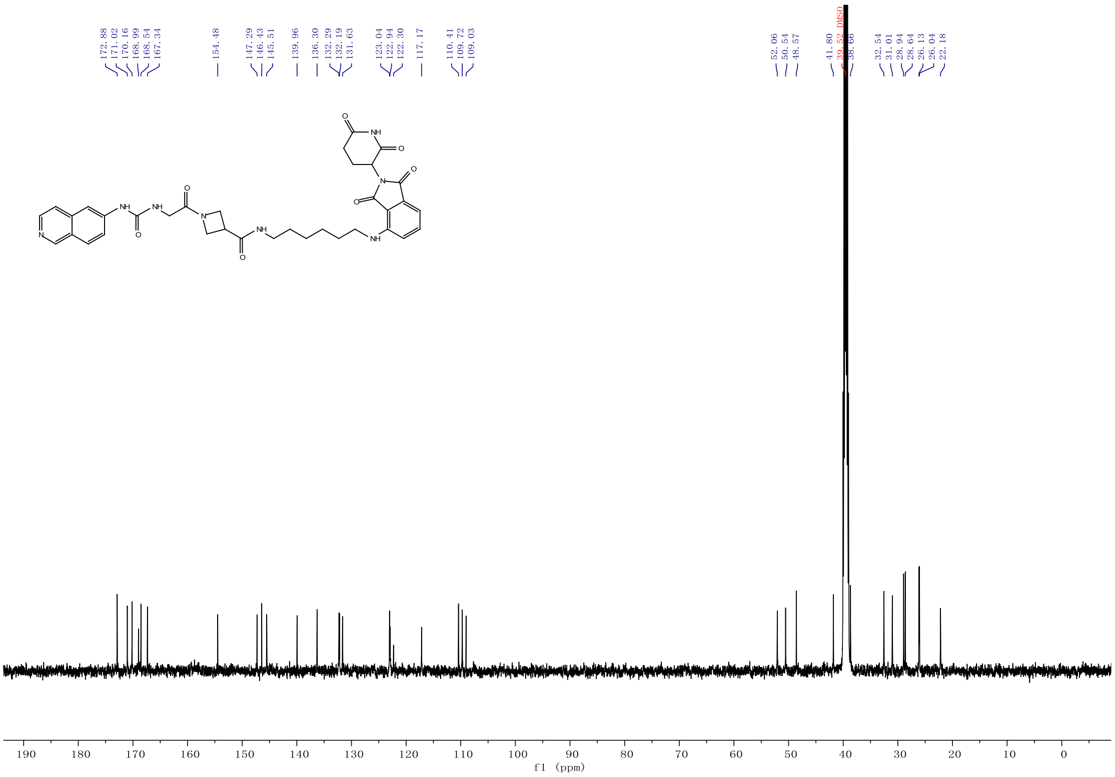

Compound 8 ^{1}H NMR

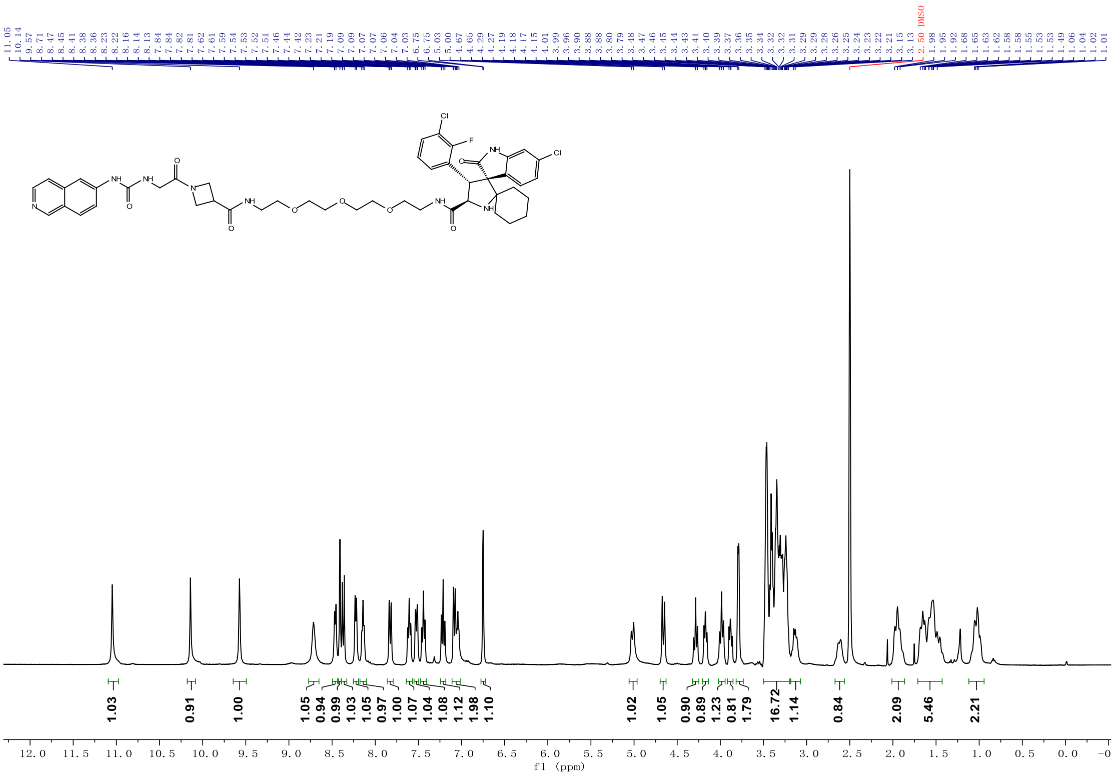

Compound 8 ^{13}C NMR

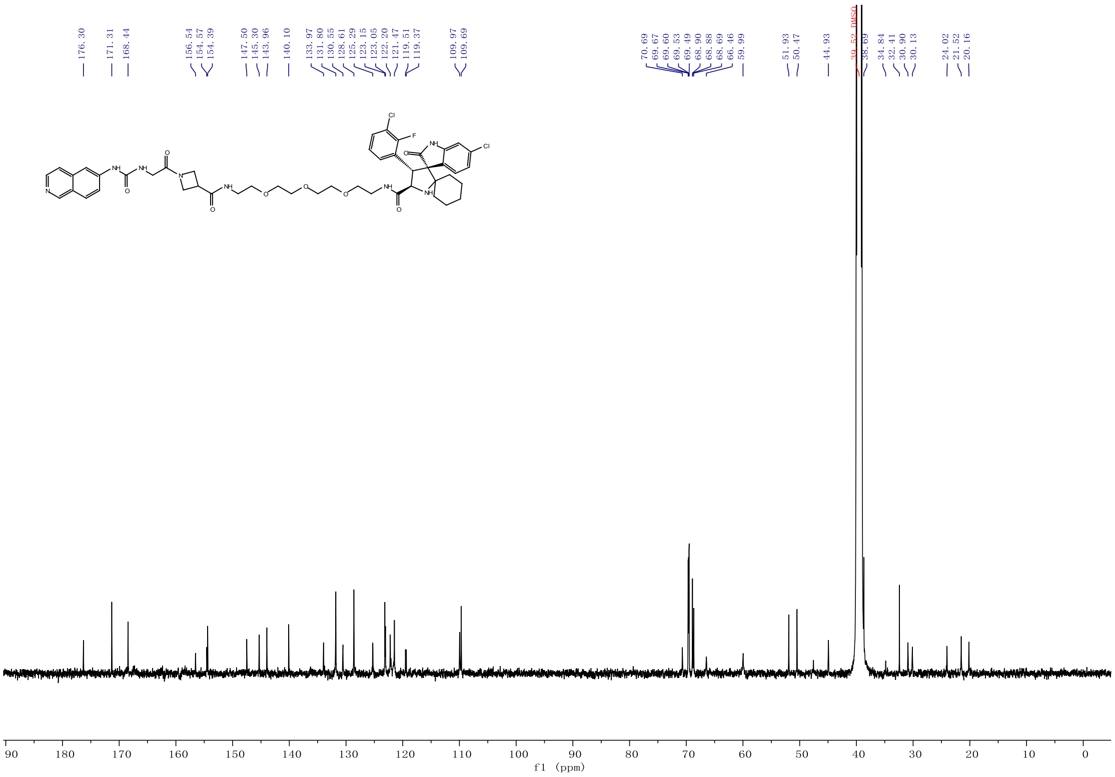

Compound 9 ^{1}H NMR

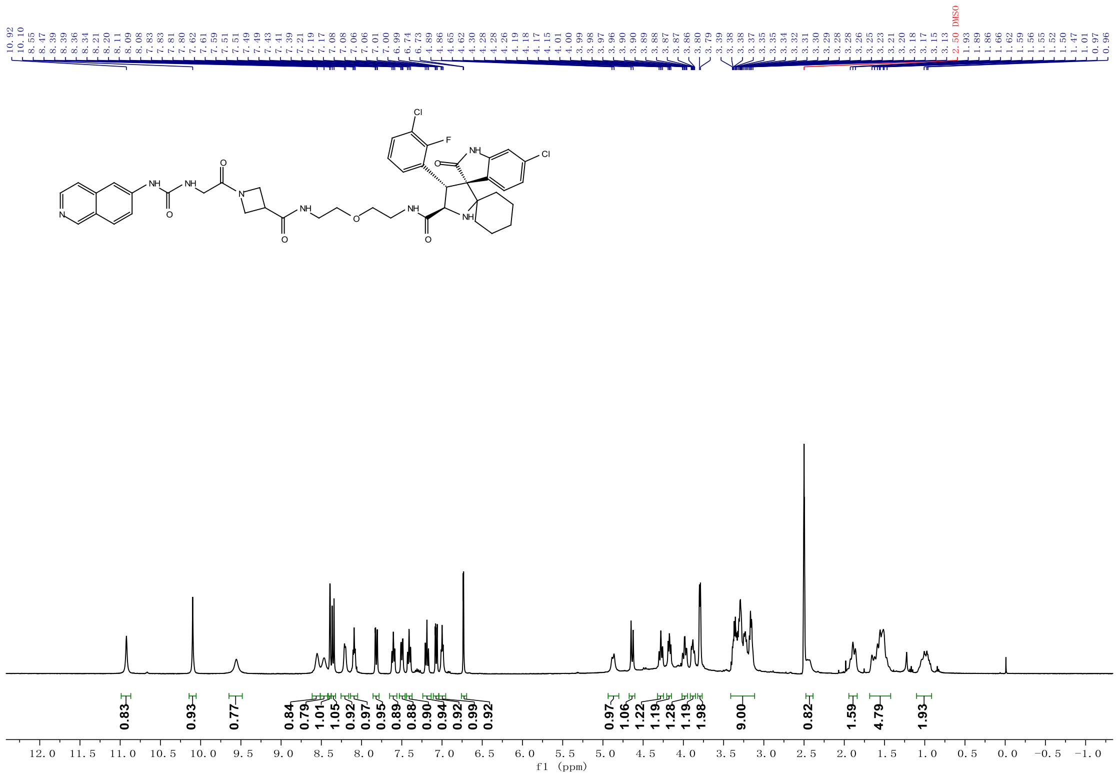

Compound 9 ^{13}C NMR

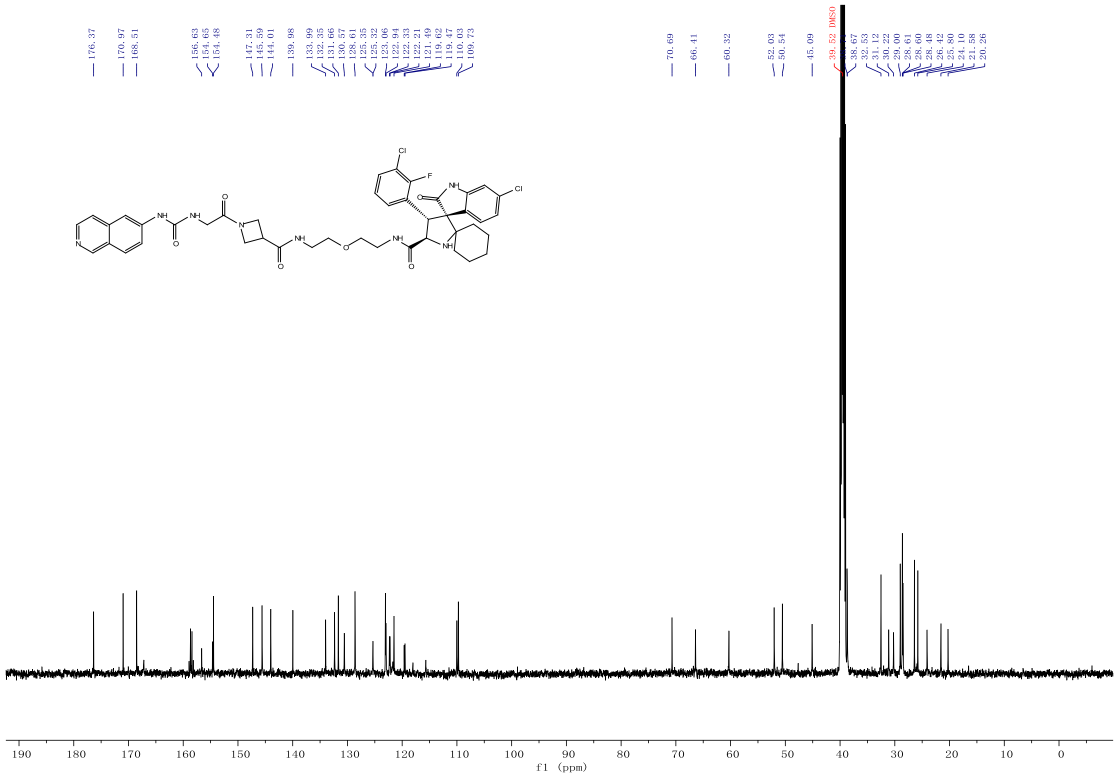

Compound 10 ^{1}H NMR

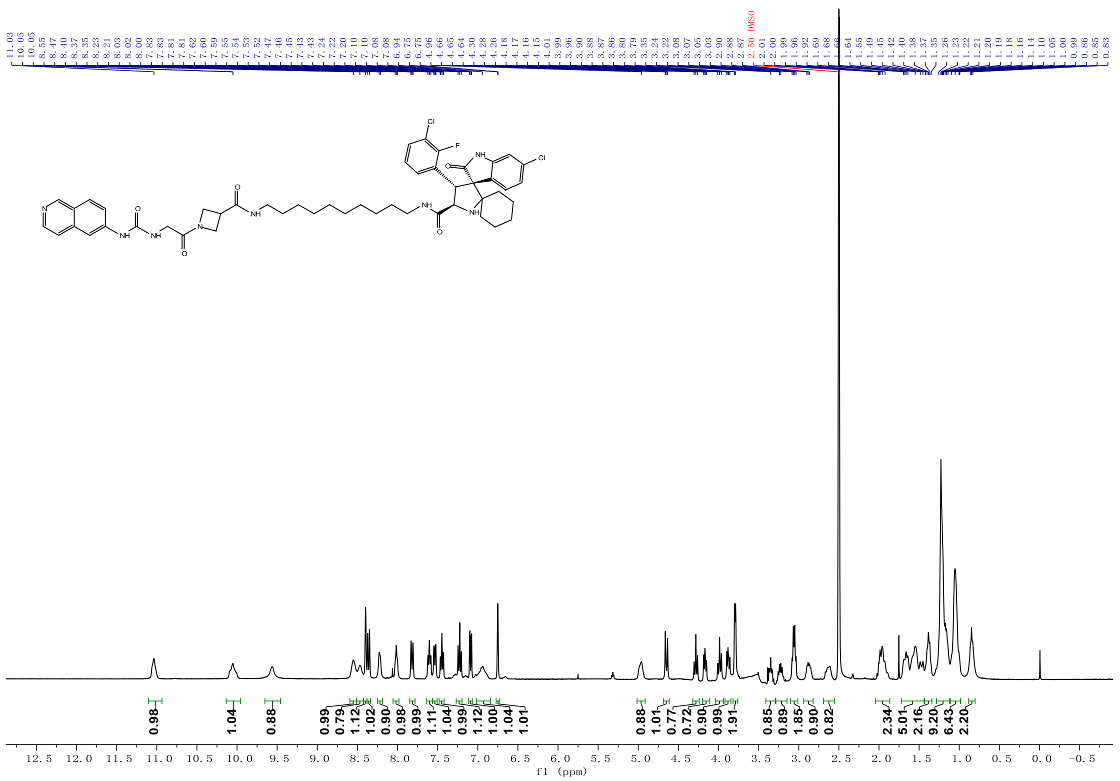

Compound 10 ^{13}C NMR

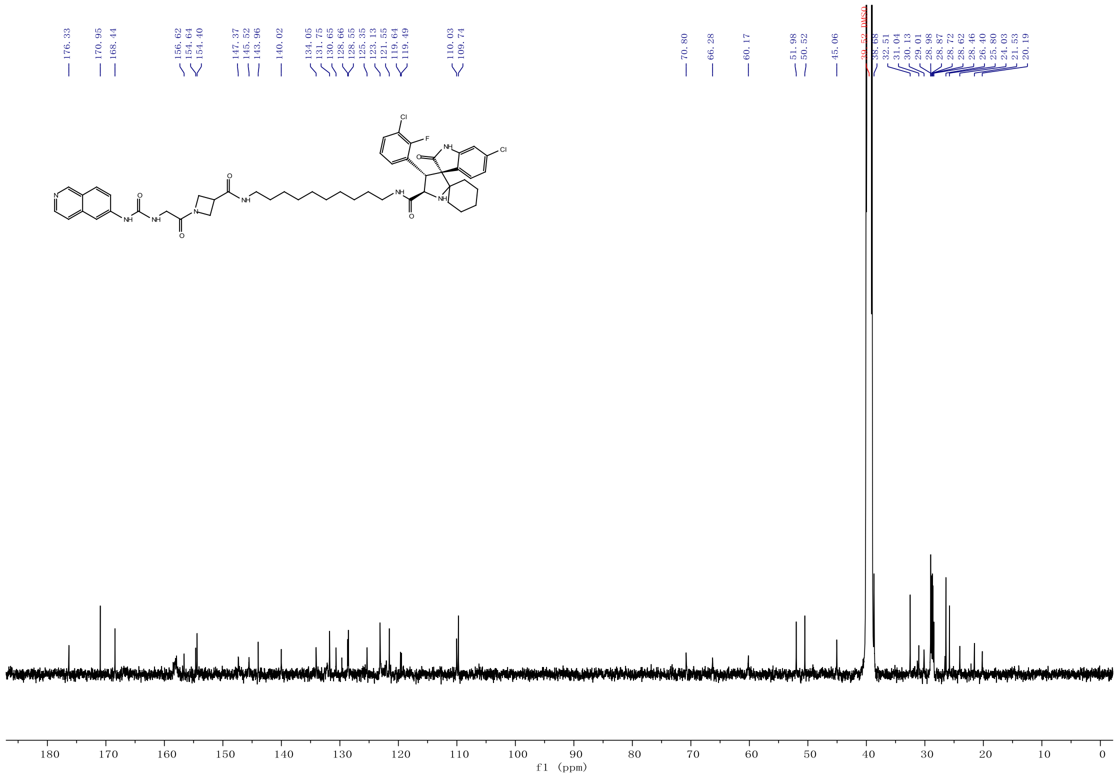

Compound 11 ^{1}H NMR

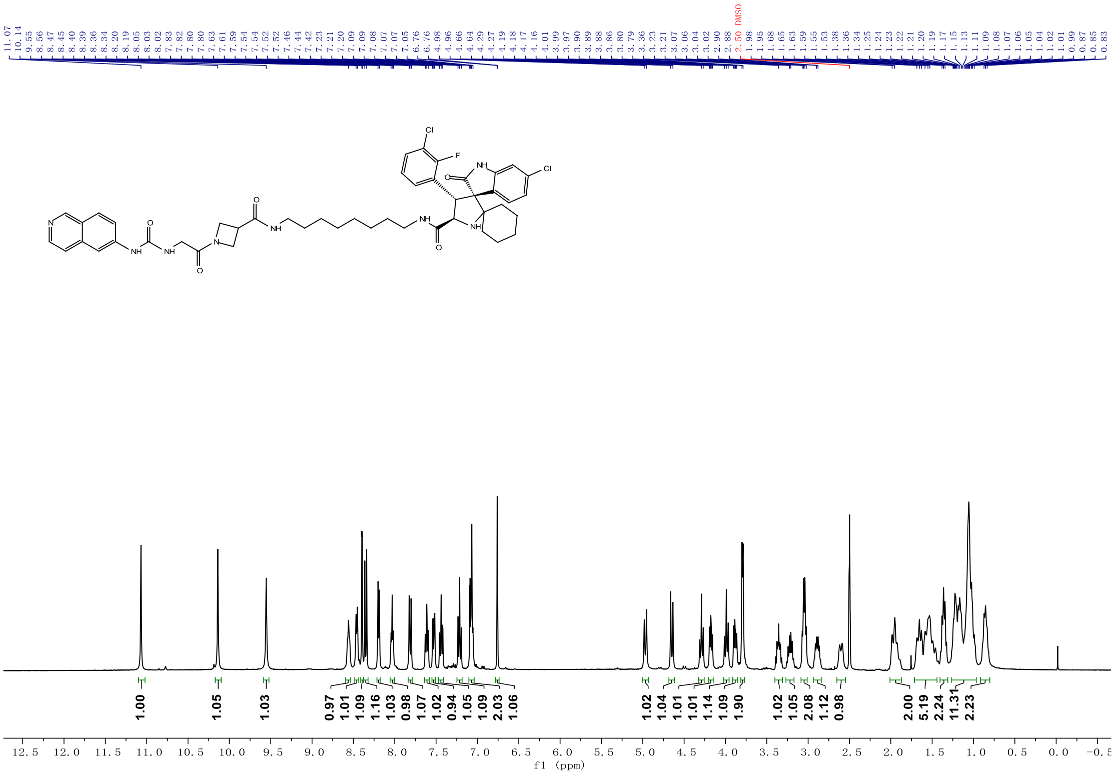

Compound 11 ^{13}C NMR

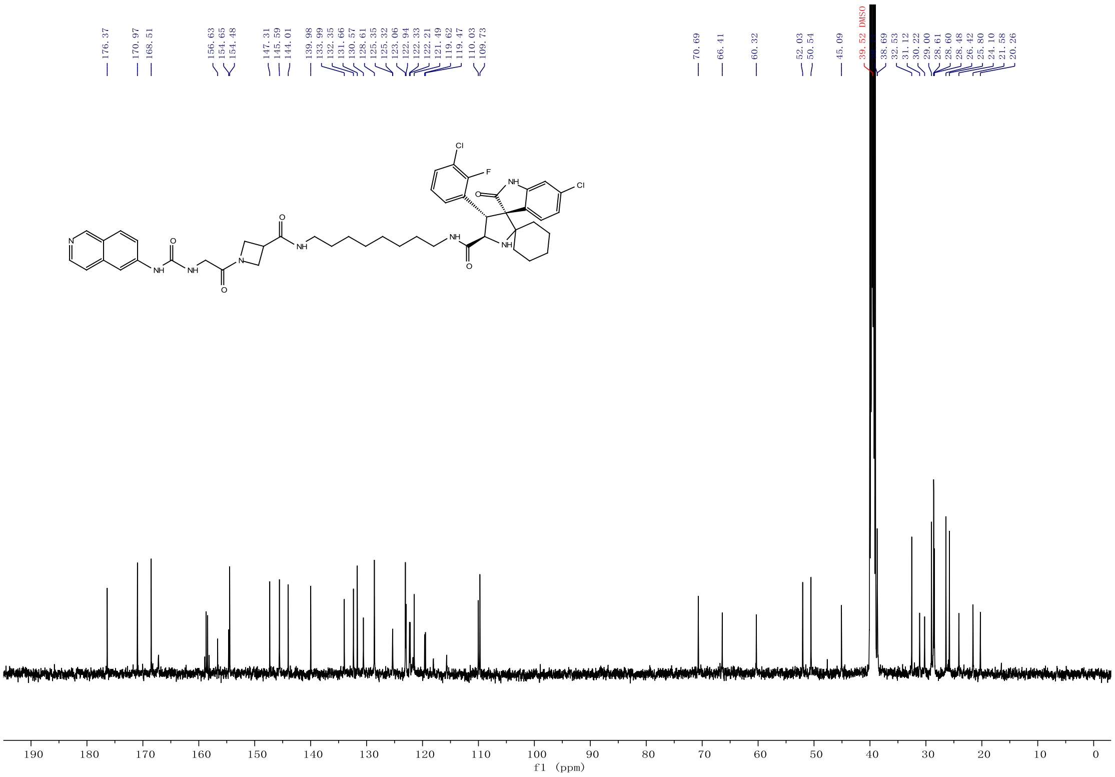

PRMT3-*i* ^{1}H NMR

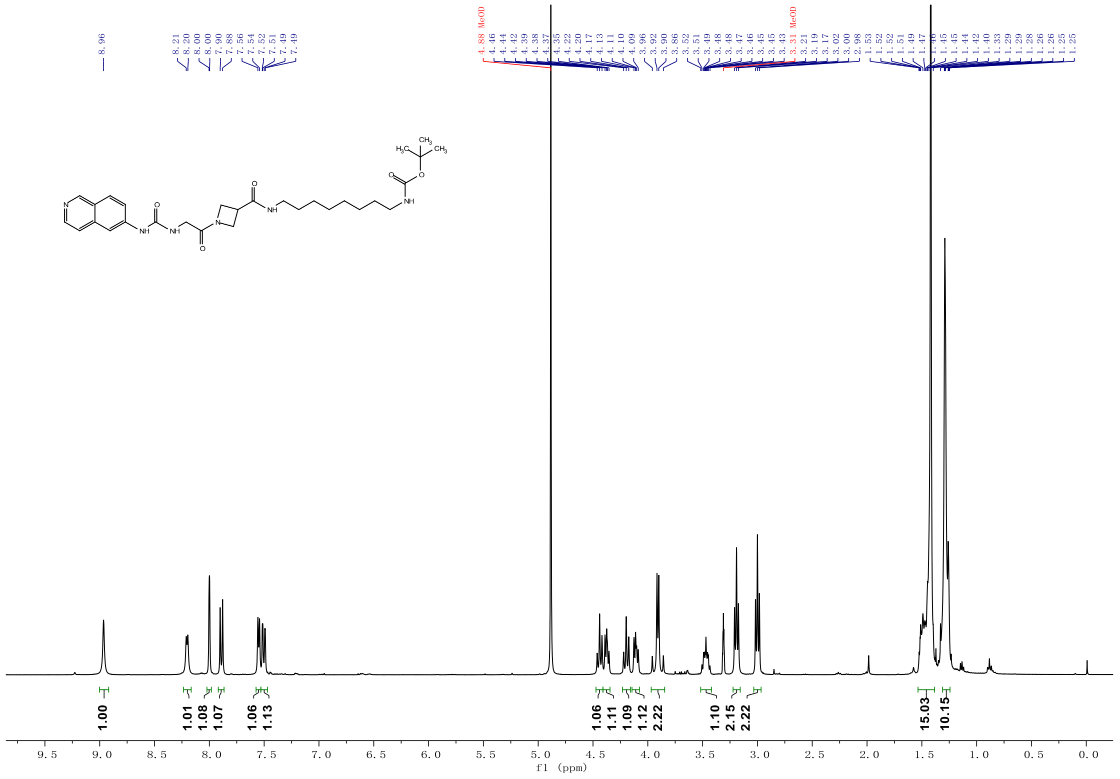

MDM2-*i* ^{1}H NMR

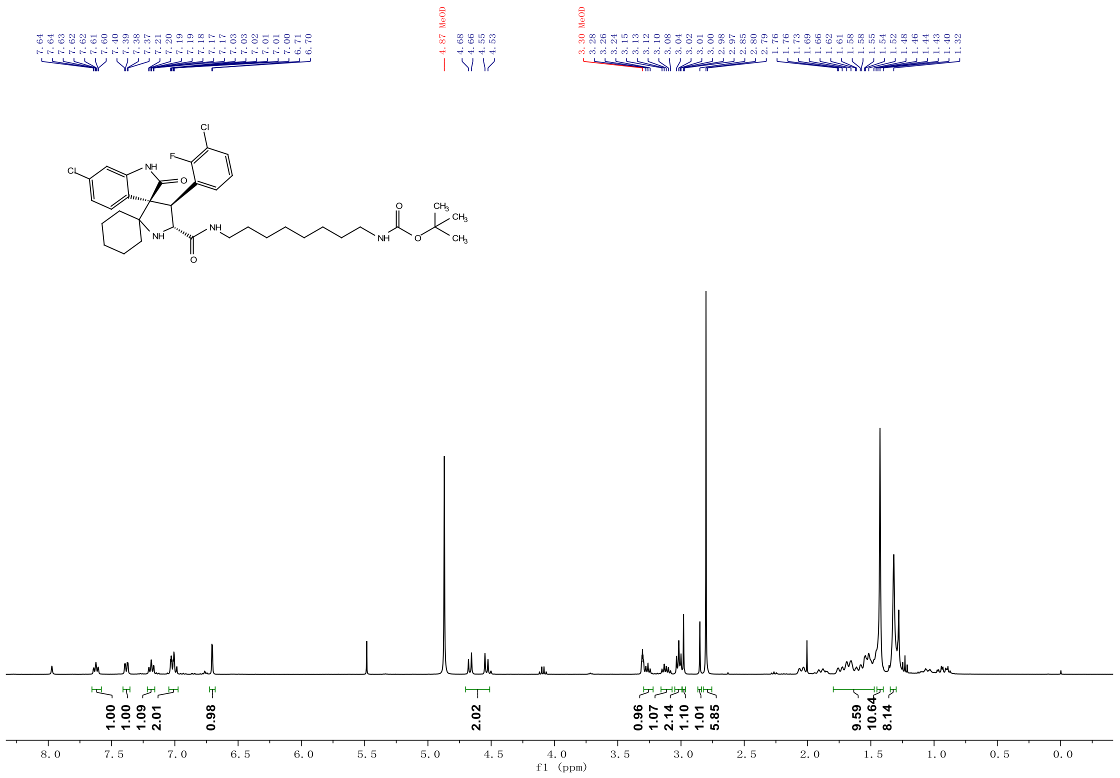

Reference

[1] Y. Liu, J. Z. Guo, Y. Liu, K. Wang, W. Ding, H. Wang, X. Liu, S. Zhou, X. C. Liu, H. B. Yang, C. Xu, W. Gao, L. Zhou, Y. P. Wang, W. Hu, Y. Wei, C. Huang, Q. Y. Lei. *Nat. Commun.* **2018**, 9(1), 4429.

[2] H. U. Kaniskan, M. M. Szewczyk, Z. T. Yu, M. S. Eram, X. B. Yang, K. Schmidt, X. Luo, M. Dai, F. He, I. Zang, Y. Lin, S. Kennedy, F. L. Li, E. Dobrovetsky, A. P. Dong, D. Smil, S. J. Min, M. Landon, J. Lin-Jones, X. P. Huang, B. L. Roth, M. Schapira, P. Atadja, D. Barsyte-Lovejoy, C. H. Arrowsmith, P. J. Brown, K. H. Zhao, J. Jin, M. Vedadi, *Angew Chem Int Edit* **2015**, *54*, 5166-5170.

[3] K. Raina, J. Lu, Y. M. Qian, M. Altieri, D. Gordon, A. M. K. Rossi, J. Wang, X. Chen, H. Q. Dong, K. Siu, J. D. Winkler, A. P. Crew, C. M. Crews, K. G. Coleman, *P Natl Acad Sci USA* **2016**, *113*, 7124-7129.

[4] A. Aguilar, W. Sun, L. Liu, J. F. Lu, D. McEachern, D. Bernard, J. R. Deschamps, S. M. Wang, *J Med Chem* **2014**, *57*, 10486-10498.
